# Dose-related immunomodulatory effects of recombinant TRAIL in the tumor immune microenvironment

**DOI:** 10.1186/s13046-023-02795-x

**Published:** 2023-08-22

**Authors:** Xupu Wang, Lizheng Wang, Wenmo Liu, Xinyao Liu, Xinyuan Jia, Xinyao Feng, Fangshen Li, Rui Zhu, Jiahao Yu, Haihong Zhang, Hui Wu, Jiaxin Wu, Chu Wang, Bin Yu, Xianghui Yu

**Affiliations:** 1 National Engineering Laboratory for AIDS Vaccine, School of Life Sciences, Jilin University, Changchun, China; 2 Hotchkiss Brain Institute, Alberta Children’s Hospital Research Institute, and the Department of Cell Biology and Anatomy, University of Calgary, Calgary, AB Canada; 3 Key Laboratory for Molecular Enzymology and Engineering, The Ministry of Education, School of Life Sciences, Jilin University, Changchun, China

**Keywords:** TRAIL, Immunoregulation, Tumor-associated macrophage

## Abstract

**Background:**

In addition to specifically inducing tumor cell apoptosis, recombinant tumor necrosis factor (TNF)-related apoptosis-inducing ligand (TRAIL) has also been reported to influence the cancer immune microenvironment; however, its underlying effects and mechanisms remain unclear. Investigating the immunomodulatory effects and mechanisms of recombinant TRAIL in the tumor microenvironment (TME) may provide an important perspective and facilitate the exploration of novel TRAIL strategies for tumor therapy.

**Methods:**

Immunocompetent mice with different tumors were treated with three doses of recombinant TRAIL, and then the tumors were collected for immunological detection and mechanistic investigation. Methodological approaches include flow cytometry analysis and single-cell sequencing.

**Results:**

In an immunocompetent mouse model, recombinant soluble mouse TRAIL (smTRAIL) had dose-related immunomodulatory effects. The optimal dose of smTRAIL (2 mg/kg) activated innate immune cells and CD8^+^ T cells, whereas higher doses of smTRAIL (8 mg/kg) promoted the formation of a tumor-promoting immune microenvironment to counteract the apoptotic effects on tumor cells. The higher doses of smTRAIL treatment promoted M2-like macrophage recruitment and polarization and increased the production of protumor inflammatory cytokines, such as IL-10, which deepened the suppression of natural killer (NK) cells and CD8^+^ T cells in the tumor microenvironment. By constructing an HU-HSC-NPG.GM3 humanized immune system mouse model, we further verified the immunomodulatory effects induced by recombinant soluble human TRAIL (shTRAIL) and found that combinational administration of shTRAIL and trabectedin, a macrophage-targeting drug, could remodel the tumor immune microenvironment, further enhance antitumor immunity, and strikingly improve antitumor effects.

**Conclusion:**

Our results highlight the immunomodulatory role of recombinant TRAIL and suggest promising therapeutic strategies for clinical application.

**Graphical Abstract:**

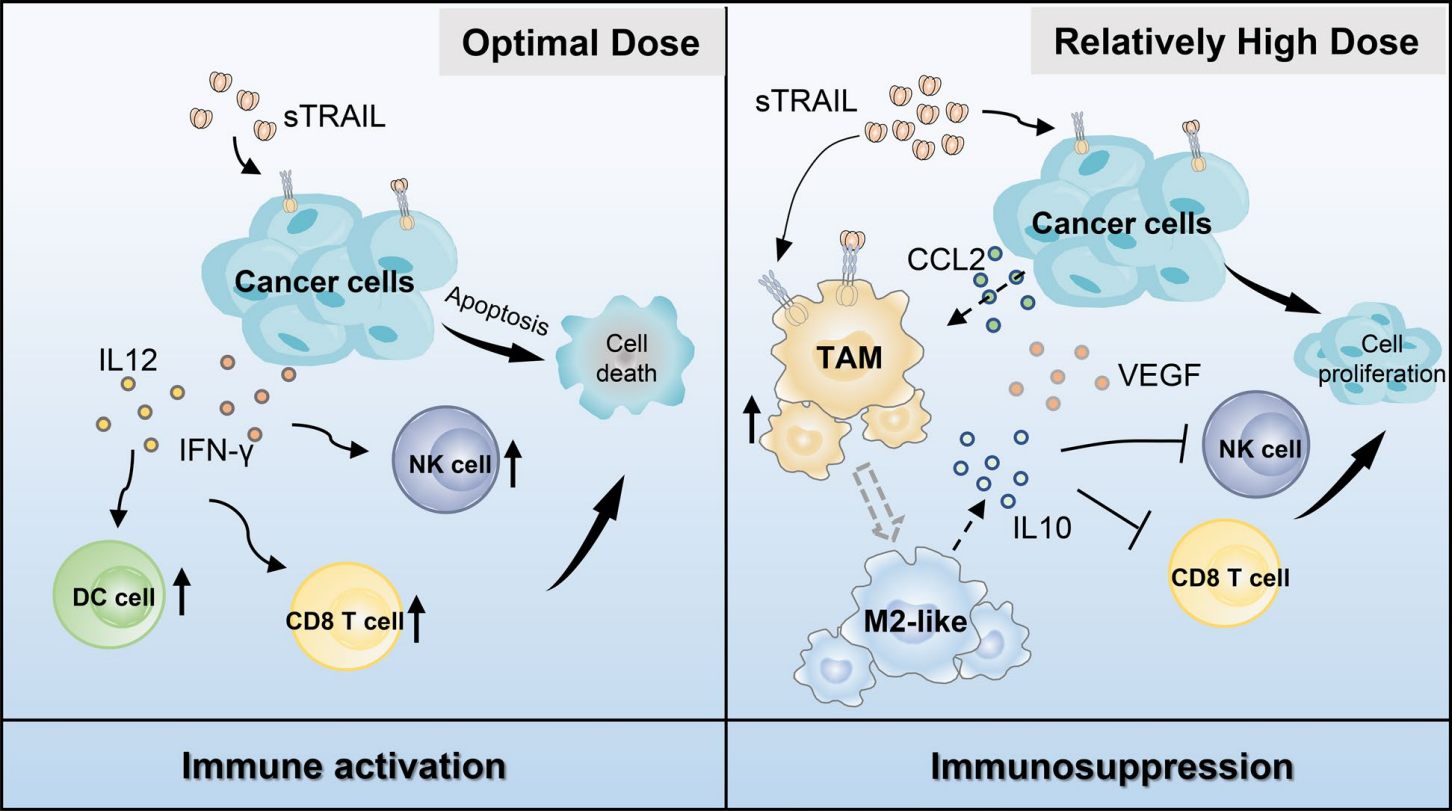

**Supplementary Information:**

The online version contains supplementary material available at 10.1186/s13046-023-02795-x.

## Background

Tumor necrosis factor-related apoptosis-inducing ligand (TRAIL) is a potential biological agent for antitumor therapy that specifically targets cancer cells to induce apoptosis [1, 2]. The therapeutic strategy in clinical studies is to use recombinant soluble TRAIL (sTRAIL) or TRAIL receptor agonists (TRAs) against TRAIL-receptors alone or in combination with chemical drugs for tumor therapy, respectively [3–6]. Clinical trials have demonstrated that systemic administration of sTRAIL is promising and safe but has no significant antitumor effect [7]. Various molecular mechanisms underlying drug resistance have been identified [8, 9]. However, further clinical research and application of TRAIL remain stagnant.

TRAIL induces apoptosis of tumor cells and is also a critical immune effector molecule in the immune system. There must be an interaction between TRAIL and the tumor immune microenvironment (TME). The TME contains an extremely diverse subset of immune cells, including T cells, B cells, NK cells, and macrophages, which help suppress tumor growth, and myeloid-derived suppressor cells (MDSCs) and regulatory T cells (Tregs), which suppress antitumor immunity [10]. TRAIL, which is expressed on monocytes, macrophages, dendritic cells (DCs), and natural killer (NK) cells, participates in the effector mechanisms of these cells and performs immune surveillance functions [11–13]. TRAIL expression in NK cells is an important mechanism through which the immune system kills cancer cells. A study also reported that intratumoral injection of sTRAIL suppresses murine hepatoma cell growth by inducing tumor-infiltrating CD4^+^CD25^+^ Treg apoptosis [14]. However, TRAIL^−/−^ mice displayed significantly decreased pancreatic cancer tumor volumes, and this tumor suppression was due to the reduction in regulatory CD4^+^ cells within the tumor [15]. Tumor-associated macrophages (TAMs) constitute a dynamic and heterogeneous population of cells within and between tumors. This heterogeneity reflects the dual function of TAMs in terms of tumor progression and outcome; they not only perform tumor-killing functions and induce antitumor responses, but also promote tumor escape and resistance to immunotherapy. This plasticity allows TAMs to rapidly acquire a range of phenotypic, metabolic, and functional profiles in various TMEs [16]. Studies have shown that the survival of MDSCs and TAMs in the TME is limited by TRAIL-R2-mediated apoptosis [17, 18]. Moreover, the endogenous TRAIL signaling pathway can recruit immunosuppressive TAMs by inducing tumor cells to secrete the cytokine CCL2, promoting an immunosuppressive microenvironment and inhibiting cytotoxic T lymphocyte (CTL) activation [19]. These findings demonstrate that TRAIL has immunomodulatory functions and an opposing regulatory role in tumor therapy. However, the immunomodulatory mechanisms of exogenous recombinant TRAIL and its role in cancer therapy remain unclear.

Because of the weak binding of human TRAIL to murine TRAIL-R [20], soluble murine TRAIL (smTRAIL) was used to evaluate the regulatory function and mechanism of recombinant TRAIL in the tumor immune microenvironment in different xenograft tumor models in immunocompetent mice. We verified the immunomodulatory effects of soluble human TRAIL (shTRAIL) in a humanized mouse immune system model. We found that recombinant TRAIL plays a dose-related bidirectional regulatory role in the TME and revealed its mechanism of action. We also explored potential strategies to enhance antitumor immunity and antitumor effects of recombinant TRAIL for cancer treatment.

## Materials and methods

### Cell lines and cell culture

The cell lines used in this study were purchased from the American Type Culture Collection (ATCC Manassas, VA, USA) and included the human colon carcinoma cell line HCT116, human colon carcinoma cell line SW620, human breast cancer cell line MCF-7, human embryonic kidney cell line HEK293T, mouse breast cancer cell line 4T1, mouse colon carcinoma cell line CT26, mouse melanoma cell line B16, mouse lymphoma cell line YAC-1, and murine macrophage cell line RAW264.7. The HCT116, SW620, 4T1, B16, and YAC-1 cell lines were cultured in RPMI 1640 (Invitrogen) supplemented with 10% heat-inactivated FBS (FBS; Gibco) and 1% penicillin/streptomycin. MCF-7, HEK293T, CT26, and RAW264.7 cells were cultured in Dulbecco's modified Eagle's medium (Invitrogen) supplemented with 10% inactivated FBS and 1% penicillin/streptomycin. Stable TRAILR-KO cells (4T1-TRAILR-KO and CT26-TRAILR-KO) were generated using CRISPR/Cas9. All cell lines were cultured in an incubator at 37 ℃ and 5% CO_2_. All cell lines were identified by short tandem repeat mapping at the start of this project. All cell lines were passaged for 2 months to ensure authenticity of cell line identity. All cells were tested for mycoplasma contamination, and all cell lines in this study were confirmed to have received treatment for mycoplasma contamination.

### Animals

Female BALB/c or C57BL/6 mice (4–6 weeks) were isolated 1 week prior to tumor implantation. A total of 5 × 10^4^ 4T1 cells, 1 × 10^5^ B16F10 cells, and 2 × 10^5^ CT26 cells in log phase were implanted subcutaneously (s.c.) into the right flank of mice. Once the tumors had grown to 100–150 mm^3^, animals were randomly assigned to different treatment groups. Each group was treated with either PBS or smTRAIL via intraperitoneal injection for eight consecutive days.

Female HU-HSC-NPG. GM3 humanized mice from Beijing Vitalstar Biotechnology Co., Ltd. were used as tumor-bearing mice. HU-HSC-NPG. GM3 mice were obtained by transferring human hematopoietic stem cells that had been steadily transferred to the GM-CSF2 and IL-3 genes into highly immunodeficient mice with NPG, a model that facilitates the expression of NK cells and macrophages. A total of 1 × 10^6^ HCT116 cells in log phase were implanted subcutaneously (s.c.) into the right flank of female BALB/c-nude mice (4–6 weeks). Once the tumors had grown to 100–150 mm^3^, the mice were treated with PBS, shTRAIL (2 mg/kg), trabectedin (0.15 mg/kg/per mouse) and a combination of shTRAIL and trabectedin. Tumor size was measured every two days using Vernier calipers, and tumor volume (mm^3^) was calculated as (length × width^2^)/2. All animals were euthanized at the end of the experiment. The differences in tumor growth were statistically significant. smTRAIL and shTRAIL are derived from soluble TRAIL protein purified by a research group in the early stages.

### Immunohistochemical (IHC) assays

Frozen sections of paraformaldehyde-fixed tumor tissues were obtained for immunohistochemical (IHC) testing. Tissue cryosections were 8–10 μm thick, dry slides for at least 10 min at 60˚C on a slide dryer. Slides were rinsed with PBS for 5 min (three times) for rehydration. After antigen repair, the tissue was blocked for 30 min using 10% goat serum in PBST. Then, the tumor tissue sections were incubated with 20 μg/mL primary goat anti-cleaved caspase-3 at selected dilutions with 1% goat serum in PBST. Place slides in a humidified chamber overnight at 4℃. After incubation with a horseradish peroxidase-conjugated anti-rabbit secondary antibody, the signal was detected using DAB. For immunofluorescence staining, the steps before the primary antibody experiment were the same as those for immunohistochemistry. For the primary antibody, tumor tissue sections were incubated with a 488-conjugated CD206 monoclonal antibody. Add mounting solution (with DAPI). Cover the slide with coverslips. The slide was dried and nail polished. The sections were scanned using confocal laser scanning microscopy (CLSM).

### Cell viability assay

Exponentially growing tumor cells or NK cells were isolated, 10^5^ cells/well were plated in a 96-well plate, and the cells were iron-coated and incubated with the protein solution of increasing concentration for 16 h. Cell viability was assessed by the MTT assay, according to previously described procedures [21].

### Preparation of single cells from the tumor

The mice were sacrificed, the tumor tissue was excised, and the tissue was cut into pieces in empty 1640 medium. Collagenase was added to a final concentration of 0.04 mg/mL, DNase I was added to 10 U/mL, digested at 37 °C for 1 h, and air-dried. A scissors 1 mL pipette tip or a 5 mL syringe was used to transfer the digested tissue and liquid to a sieve plate prefilled with 10 mL R10. The tumor tissue was ground with a 5 mL syringe pusher, the liquid outside the screen was transferred to a 50 mL centrifuge bucket, and 5 mL R10 was used for cleaning the screens and dishes. The prepared cell suspension was passed through a 200-mesh sieve into a new centrifuge bucket and centrifuged at 350 g for 5 min. The supernatant was discarded and the cells were resuspended in medium containing 10% serum for cell counting at 10^6^/tube for each group.

### Flow cytometry

Single-cell suspensions of the tumors were prepared as described previously. For surface staining, cells were kept for 30 min at 4 ℃ and blocked with TruStain FcX™ (anti-mouse CD16/32) antibody prior to staining. For intracellular staining, fixation and permeabilization were performed according to the instructions, followed by intracellular staining. The samples were acquired on a FACS Verse instrument (Becton Dickinson) and analyzed using FlowJo version 10.

### RNA extraction and real-time RT–qPCR

Total RNA from frozen tumor tissues or cells was extracted using a TransZol™ Up Plus RNA Kit. Complementary DNA was generated using the PrimeScript™ 1^st^ Strand cDNA Synthesis Kit, according to the manufacturer’s instructions. The SYBR green method was used for RT-qPCR on the Real-Time Detection System (Bio-Rad). The relative expression was normalized to that of β-actin in each murine sample. Relative expression was normalized to that of GAPDH in each human sample.

### Immunoassays for secreted chemokines and cytokines

The tumor tissue stored at -80℃ was ground at low temperature, the PBS-RIPA grinding buffer was PBS-RIPA (containing protease inhibitors), the tissue was lysed on ice for 30 min and centrifuged at 12,000 rpm for 5 min, and the supernatant was collected. The detection and quantification of secreted chemokines and cytokines were performed as described by the manufacturer (LEGENDplex™ Mouse Inflammation Panel (13-plex) with Filter Plate). Samples were analyzed via flow cytometry and the results were analyzed using the analysis software LEGENDplex v8.0, provided by BioLegend.

### Single-cell RNA sequencing (scRNA-Seq) by 10 × Genomics

Cell suspensions were loaded into chromium microfluidic chips with 3' v3 chemistry and barcoded using a 10 × Chromium Controller (10 × Genomics). RNA from the barcoded cells was subsequently reverse transcribed, and a sequencing library was constructed according to the instructions for the Chromium Single Cell 3' v3 kit (10 × Genomics). Sequencing was performed using the Illumina platform.

### Generation and analysis of single-cell transcriptomes

Raw reads were demultiplexed and mapped to the reference genome using the 10X Genomics Cell Ranger pipeline using default parameter values. General downstream single-cell analysis was performed using Cell Ranger and Seurat [22, 23]. SCTransform was used to normalize the data, and harmony was used to correct the data batch effects [24]. Marker genes (expressed in at least 25% of cells in the cell populations) for clusters were determined using the FindAllMarker function. Differentially expressed genes (DEGs) between groups were identified using FindMarker. Gene set enrichment analysis (GSEA) of the smTRAIL-treated groups compared to the PBS group was performed using the R package GSEA and clusterProfiler. The Monocle3 R package was used for the trajectory analysis of cell clusters [25–28]. The density plot of representative gene expression was visualized using the R package Nebulosa [29].

### Retroviral transduction

Lentivirus packaging was carried out in HEK293 cells according to previously described methods. The obtained virus-infected 4T1 cells and CT26 cells were treated with the pro-infection reagent polybrene, and then screened by puromycin to obtain 4T1-TRAIL-R-KO and CT26-TRAIL-R-KO cells. On day 7 of culture, cells were harvested and transduced cells were identified by mouse TRAILR expression via flow cytometry.

### Isolation of primary NK cells

Spleens were obtained after the mice were sacrificed. A single-cell suspension was prepared from the spleen using a manual method. After counting, the NK cells were sorted using a mouse NK cell isolation kit. The specific operational steps were carried out according to the method provided by the kit. The isolated cells were confirmed by gating CD49b^+^/CD3^−^ cells. The obtained NK cells were cultured in RPMI 1640 medium containing 10% serum and 200 U/ml recombinant mouse IL-2 for 16 h before use in the assays.

### NK cell cytotoxicity assay

Prepare 100 nM protein stock solutions for smTRAIL and prepare protein solutions by tenfold dilution. The NK cells were incubated with serial dilutions of smTRAIL proteins for 16 h at 37 °C. After centrifugation, the supernatant was removed, and NK cells were resuspended in the culture medium and counted. NK cells were incubated with YAC-1 cells at E:T ratios of 10:1 or 3:1. After 4 h, a lactate dehydrogenase cytotoxicity assay kit was used to detect NK cell cytotoxicity. Cytotoxicity or death rate (%) = (absorbance of treated sample − absorbance of sample control well) / (absorbance of maximum enzyme activity of cells − absorbance of sample control well) × 100.

### Acquisition and polarization of macrophages

RAW264.7 cells were polarized towards M1 with the addition of LPS (100 ng/ml), and towards M2 with the addition of IL-4 (20 ng/ml) and IL-10 (20 ng/ml). After 24 h of stimulation, the expression of M1-like macrophage polarization-related genes (iNOS, TNF-α, and CD80) and M2-like macrophage polarization-related genes (MRC-1, and Arg-1) was detected by real-time qPCR. Meanwhile, the expression of M1-like and M2-like marker proteins after polarization was examined by flow cytometry. M1-like macrophages upregulated the expression of CD86 and M2-like macrophages upregulated the expression of CD206.

### Data analysis and statistics

All statistical analyses were performed using GraphPad Prism for data analysis. Comparisons of three or more groups were calculated using one-way analysis of variance (ANOVA) and, where indicated, unpaired or paired two-tailed Student's t -test. Statistical significance was considered when the *P* value was < 0.05. Numerical data were tested for normal distribution using the Kolmogorov–Smirnov test. Normally distributed data are presented as the mean ± standard deviation. In vivo pilot studies were conducted to estimate the sample size required to ensure adequate power. Age- and sex-matched animals were randomly assigned to the experimental conditions during the experiment. Data collection and analysis were not performed regardless of the experimental conditions. Data exclusion was not performed.

## Results

### Antitumor efficacy of smTRAIL is dose-independent in immunocompetent mouse models

**Fig. 1:**
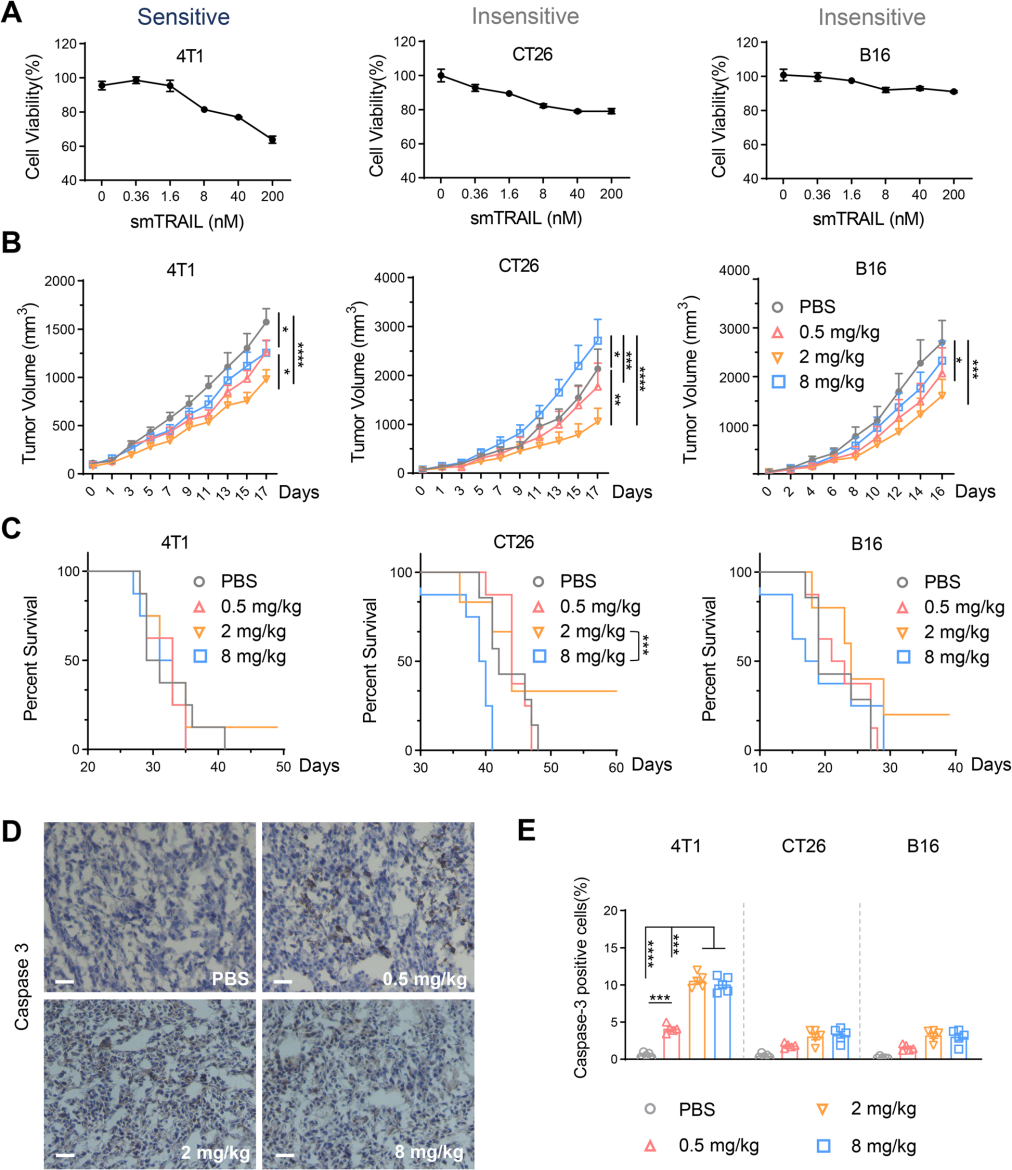
smTRAIL induces tumor cell apoptosis and antitumor responses in mouse models. **A** Murine cancer cell lines were treated with the indicated concentrations of smTRAIL for 16 h, and cell viability was determined by MTT assay. **B** Tumor growth curves and **C** survival curves with three concentrations of smTRAIL in different tumor-bearing models (*n* = 8). BALB/c mice were subcutaneously (s.c.) inoculated with 5 × 10^4^ 4T1 cells or 2 × 10^5^ CT26 cells, whereas C57BL/6 mice were s.c. inoculated with 1 × 10^5^ B16 cells in the right flanks, and smTRAIL was injected intraperitoneally (i.p.) for eight consecutive days. **D** Detection of caspase-3 in tumor tissues. Mice treated with different doses of smTRAIL were euthanized on day 20. Activation of caspase-3 was detected by immunohistochemistry. Scale bars, 50 μm. **E** Data indicate the percentage of caspase-3 positive cells in tissue sections, with at least 5 images being analyzed. A two-way ANOVA test was used to compare the tumor growth responses. *P < 0.05; **P < 0.01; ***P < 0.001 and ****P < 0.0001

TRAIL induces tumor cell apoptosis by binding to TRAIL-R. Because of the differences between human and murine TRAIL-R and the poor affinity of human TRAIL for murine TRAIL-R, we expressed soluble murine TRAIL (smTRAIL) for experimental evaluation [30]. To evaluate the cytotoxic activity of smTRAIL on tumor cells in vitro and in vivo, we first determined the cytotoxic effect of smTRAIL on three murine tumor cell lines by MTT assay. smTRAIL did not induce obvious cell death in CT26 and B16 cells, whereas 4T1 cells were more sensitive to smTRAIL (Fig. 1A). The sensitive cell line 4T1 and the non-sensitive cell lines CT26 and B16 were selected to establish tumor-bearing mouse models and to evaluate the antitumor effect of smTRAIL in vivo. In a phase I dose-escalation study [6], three different concentrations of smTRAIL were administered by intraperitoneal injection for eight consecutive days. The results showed that the group that received a protein concentration of 0.5 mg/kg significantly inhibited tumor growth in both the 4T1 and B16 models. Interestingly, the 2 mg/kg group exhibited the best inhibition of tumor growth in mice in all three tumor models and significantly prolonged survival compared with the 8 mg/kg group in the CT26 model, while the 8 mg/kg group exhibited weak antitumor activity in both the 4T1 and B16 models and even significantly promoted tumor growth compared to the PBS group in mice with CT26 tumors (Fig. 1B and C). Tumor tissues were obtained for analysis by detecting the expression of caspase-3. Caspase-3 was readily detected in 4T1 tumor tissues (Fig. 1D and E), whereas there was only very weak expression of caspase-3 in CT26 and B16 tumor tissues (data not shown). These results suggest that the tumor inhibitory activity of smTRAIL on CT26 and B16 cells in vivo does not significantly correlate with its ability to induce tumor cell apoptosis, indicating that smTRAIL has other roles in tumor treatment.

### smTRAIL exerts dose-related bidirectional immunoregulatory effects in both apoptosis-sensitive and apoptosis-insensitive tumor models

**Fig. 2:**
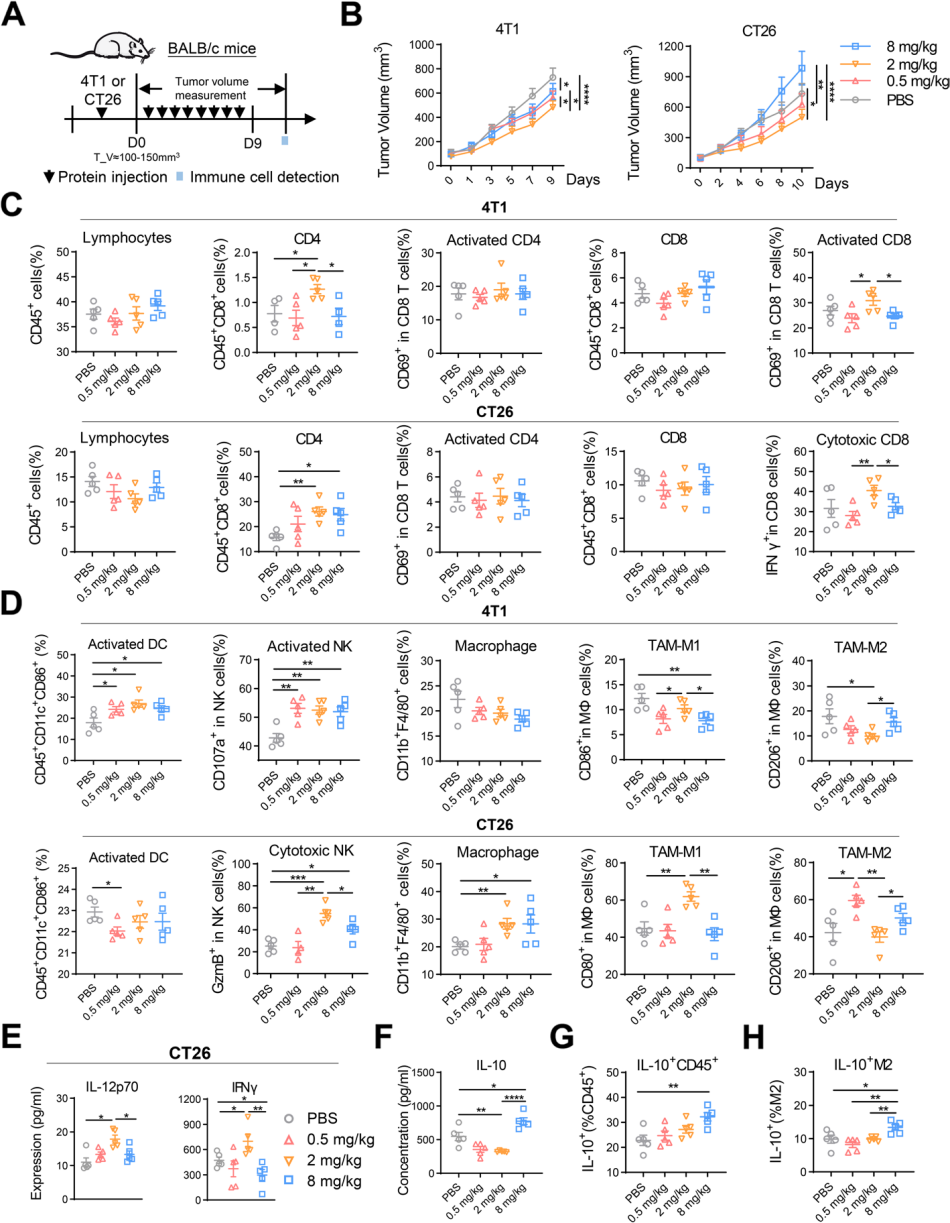
Different doses of smTRAIL have different immunomodulatory functions. **A** Experimental layout. Three days after the treatment, 4T1 and CT26 tumor tissues were obtained for immune cell analysis by flow cytometry. **B** Short-term tumor growth curve of smTRAIL treatment (*n* = 13). **C** 4T1 and CT26 tumors analyzed by flow cytometry to calculate the percentages of intratumoral lymphocytes (CD45^+^), CD4^+^ T cells (CD45^+^CD4^+^), activated CD4^+^ T cells (CD45^+^CD4^+^CD69^+^), CD8^+^ T cells (CD45^+^CD8^+^), activated CD8^+^ T cells (CD45^+^CD8^+^CD69^+^/IFNγ^+^), **D** activated DC cells (CD45^+^CD8^+^CD69^+^), activated NK cells (CD3^−^CD49b^+^CD107a^+^/GzmB^+^), TAMs (CD45^+^F4/80^+^CD11b^+^), M1-TAMs (CD45^+^F4/80^+^CD11b^+^CD86^+^), and M2-TAMs (CD45^+^F4/80^+^CD11b^+^CD206^+^) (*n* = 5). **E** CT26 tumor tissues were obtained and the levels of IL-12p70 and IFNγ were analyzed (*n* = 5). **F** The expression of IL-10 in CT26 tissues was analyzed by ELISA (*n* = 5). **G** The percentages of IL-10^+^CD45^+^ were analyzed (*n* = 5). **H** The percentages of IL-10^+^ M2-TAMs were analyzed (*n* = 5). A two-way ANOVA test was used to compare the tumor growth curves. One-way ANOVA was performed to calculate the significant differences between groups, followed by LSD analysis. *P < 0.05; **P < 0.01; ****P < 0.0001

Our results showed that smTRAIL could effectively inhibit both sensitive and non-sensitive tumor cells in vivo, but a higher dose of smTRAIL appeared to lose its antitumor effect. In addition to apoptosis, immunomodulation of TRAIL also affects tumor growth. To evaluate the immunomodulatory effects of exogenous recombinant TRAIL in the tumor immune microenvironment, we established two tumor models, 4T1 and CT26, in BALB/c mice and treated them with three concentrations of smTRAIL. Immune cells were detected in the tumors within 96 h of the last treatment (Fig. 2A). Consistent with the results of long-term monitoring, 2 mg/kg of smTRAIL was the most effective in inhibiting tumor growth, and 8 mg/kg of smTRAIL promoted tumor growth compared to the other smTRAIL doses in the CT26 tumor model, even during short-term monitoring (Fig. 2B).

Immune cells in the tumor tissues of the 4T1 and CT26 tumor-bearing models were assayed using flow cytometry. As shown in Fig. 2C, administration of smTRAIL did not change the number of intratumoral CD45^+^ immune cells, activated CD4^+^ T cells, or CD8^+^ T cells in either model. The 2 mg/kg smTRAIL group showed a significant increase in the number of CD4^+^ T cells in the 4T1 model compared to the other groups. In the CT26 model, both 2 mg/kg and 8 mg/kg smTRAIL significantly increased the percentage of CD4^+^ T cells. Interestingly, we found that the percentage of activated CD8^+^ T cells in the 2 mg/kg smTRAIL group was significantly higher than those in the 0.5 mg/kg smTRAIL group and 8 mg/kg smTRAIL groups in both models (Fig. 2C). By assaying innate immune cells, treatment with 2 mg/kg smTRAIL significantly increased the number of dendritic cells (DCs) in the CT26 model and the percentage of activated DCs in the 4T1 model (Fig. 2D and Fig. S1A). We found that in the 4T1 model, the number of NK cells in the 8 mg/kg smTRAIL group was significantly inhibited, and that smTRAIL treatment significantly increased the activation of NK cells, whereas in the CT26 model, the number of cytotoxic NK cells in the 2 mg/kg smTRAIL group was significantly higher than that in the other groups (Fig. 2D and Fig. S1A). However, there were some differences in macrophage results between the two models. In a CT26 mouse model, smTRAIL treatment significantly increased the number of intratumoral macrophages. We also found that the 2 mg/kg smTRAIL group had a significantly increased number of intratumoral M1 macrophages compared with the 8 mg/kg smTRAIL and PBS groups, whereas the 0.5 mg/kg and 8 mg/kg smTRAIL groups had a clearly increased number of intratumoral M2 macrophages compared with the 2 mg/kg smTRAIL groups. However, when only the treatment groups were compared, the percentage of M1 macrophages in the 2 mg/kg smTRAIL group was significantly higher than those in the 0.5 mg/kg smTRAIL and 8 mg/kg smTRAIL groups, whereas the percentage of M2 macrophages in the 8 mg/kg smTRAIL group was significantly higher than that in the 2 mg/kg smTRAIL group in both tumor models (Fig. 2D). Treatment with smTRAIL increased the number of monocytic myeloid-derived suppressor cells (M-MDSCs) in the 4T1 model (Fig. S1B), but had little effect on immunosuppressive Tregs and PMN-MDSCs in either model (Figs. S1B and C).

We also used tumor tissues from a 4T1 tumor-bearing model for RNA sequencing. Due to the weak therapeutic effect and immunoregulatory ability of 0.5 mg/kg smTRAIL, only the 2 mg/kg and 8 mg/kg smTRAIL groups were selected. The robustness of CIBERSORTx [31] in predicting cell type-specific gene expression profiles from The Cancer Genome Atlas (TCGA) and Gene Expression Omnibus (GEO) datasets was tested using our scRNA-seq dataset (Fig. S2A). The changes in the immune cells after smTRAIL treatment were consistent with the flow cytometry results. GO analysis of the RNA-seq data showed that the 2 mg/kg smTRAIL group could activate NK cell-mediated immunity and positively regulate the activation and proliferation of T cells, whereas the regulation of the 8 mg/kg smTRAIL group in tumor tissues was related to DNA replication (Fig. S2B). Genes associated with immune activation were significantly downregulated in the 8 mg/kg smTRAIL-treated group (Fig. S2C).

To explore the possible reasons for these immune cell changes, we examined the expression levels of related cytokines and chemokines in the TME. Analysis of protein expression levels revealed that 2 mg/kg smTRAIL significantly increased the expression of IL-12p70 and IFNγ in tumors, exerting an immune-activating function (Figs. S1F, G and Fig. 2E). mRNA expression analysis showed that after treatment with 8 mg/kg of smTRAIL, *Il-10* Th1 inhibitor and vascular endothelial growth factor (*Vegf*) expression significantly increased in both models (Figs. S1D and E). Analysis of protein expression levels also revealed that 8 mg/kg smTRAIL significantly increased IL-10 expression in the TME (Fig. 2F and Fig. S1G). Cytokine-secreting cells were also analyzed in the CT26 model, and we found that the percentage of IL-10^+^ lymphocytes in the 8 mg/kg smTRAIL group was significantly higher than that in the control group (Fig. 2G), whereas the expression of IL-10 was particularly evident in M2 macrophages (Fig. 2H). Together, these results show that smTRAIL has an immunomodulatory function in the TME, with treatment with 2 mg/kg of smTRAIL activating innate immune cells and CD8^+^ T cells to suppress tumor growth, whereas treatment with 8 mg/kg of smTRAIL was less effective or even promoted tumor growth, probably due to the formation of a tumor immunosuppressive environment associated with M2 macrophages and the secretion of immunosuppressive cytokines such as IL-10.

### Immunomodulatory effects of smTRAIL analyzed by single-cell RNA sequencing in the CT26 model

To further clarify the immunomodulatory effects and mechanism of action of smTRAIL in the tumor immune microenvironment, we performed scRNA-seq analysis of tumor tissues from the CT26 model. After passing the quality control metrics, 22,945 cell transcriptomes from tumors were retained for subsequent analysis, of which 7192 cells originated from the PBS control group, 7795 from the 2 mg/kg smTRAIL group, and 7958 from the 8 mg/kg smTRAIL group. All major tumor-infiltrating cell types (macrophages, carcinoma-associated fibroblasts (CAFs), T cells, NK cells, endothelial cells, and neutrophils) were present in the smTRAIL-treated and control groups (Fig. S3A). Tumor cells were identified by *Baiap2l1* and *Rpl39l*, macrophages were identified by the expression of *Adgre1* and *Csf1r*, CAFs were marked by *Col1a1* and *Bgn*, T cells expressed the T-cell receptor (TCR) signaling mediators *Cd3d* and *Cd3e*, NK cells were identified by *Ncr1* and *Nkg7* expression, neutrophils were positive for *Cxcr2* and *S100a9* expression, and endothelial cells were positive for *Cdh5* and *Pecam1* (Figs. S3B and C). For antitumor immune effector cells, we first classified the three identified sub-populations of NK cells using previous literature as CC chemokine-producing NK (*Ccl5*, *Ccl3*, *Ccl4*) [32], cytotoxic NK (*Gzmc*, *Gzmd*, *Gzme*, and *Gzmq*), and proliferative NK (*Mki67*) cells (Figs. S3D and E). By comparing the differential infiltration of NK cell subtypes between the smTRAIL-treated groups and the control, we found that CC chemokine-producing NK cells were markedly enriched, but cytotoxic NK cells were reduced in the 8 mg/kg smTRAIL-treated group compared with the other two groups, whereas proliferative NK cells were enriched in the 2 mg/kg smTRAIL-treated group compared with the other two groups (Fig. S3F). We classified five subpopulations of T cells: progenitor-exhausted CD8^+^ (*Ly6c2*, *Ccl5*, *Gzmk*, and *Ctla2a*), Pdcd1^high^ CTL (*Cd8a*, *Gzmb*, and *Pdcd1*), activated naïve T (*Il7r* and *cd69*), proliferative CD8^+^ (*Hist1h1b*, *Mki67*, and *Tubb5*), and Tregs (*Il7r*, *Foxp3*, and *Ctla4*) (Figs. S3G and H) [33]. By analyzing the percentages of T-cell subclusters in the PBS, 2 mg/kg smTRAIL, and 8 mg/kg smTRAIL groups, we found that the CD8^+^ proliferative cell subset exhibited differences with proportions of 10.97%, 20.55%, and 13.98%, respectively (Figs. S3I and J). This indicates that smTRAIL treatment can enhance the infiltration of proliferative *Pdcd1*^high^ CTL into the TME, but the infiltration ratio of the 8 mg/kg smTRAIL group was inhibited compared to that of the 2 mg/kg smTRAIL group.

These results further confirmed that smTRAIL has an immunomodulatory role in the TME and showed that 2 mg/kg smTRAIL treatment can effectively activate NK cells and CD8^+^ T cells in the TME, whereas 8 mg/kg smTRAIL treatment restrained this activation effect.

### Regulatory effect of smTRAIL on NK cells in immunodeficient mouse models

**Fig. 3:**
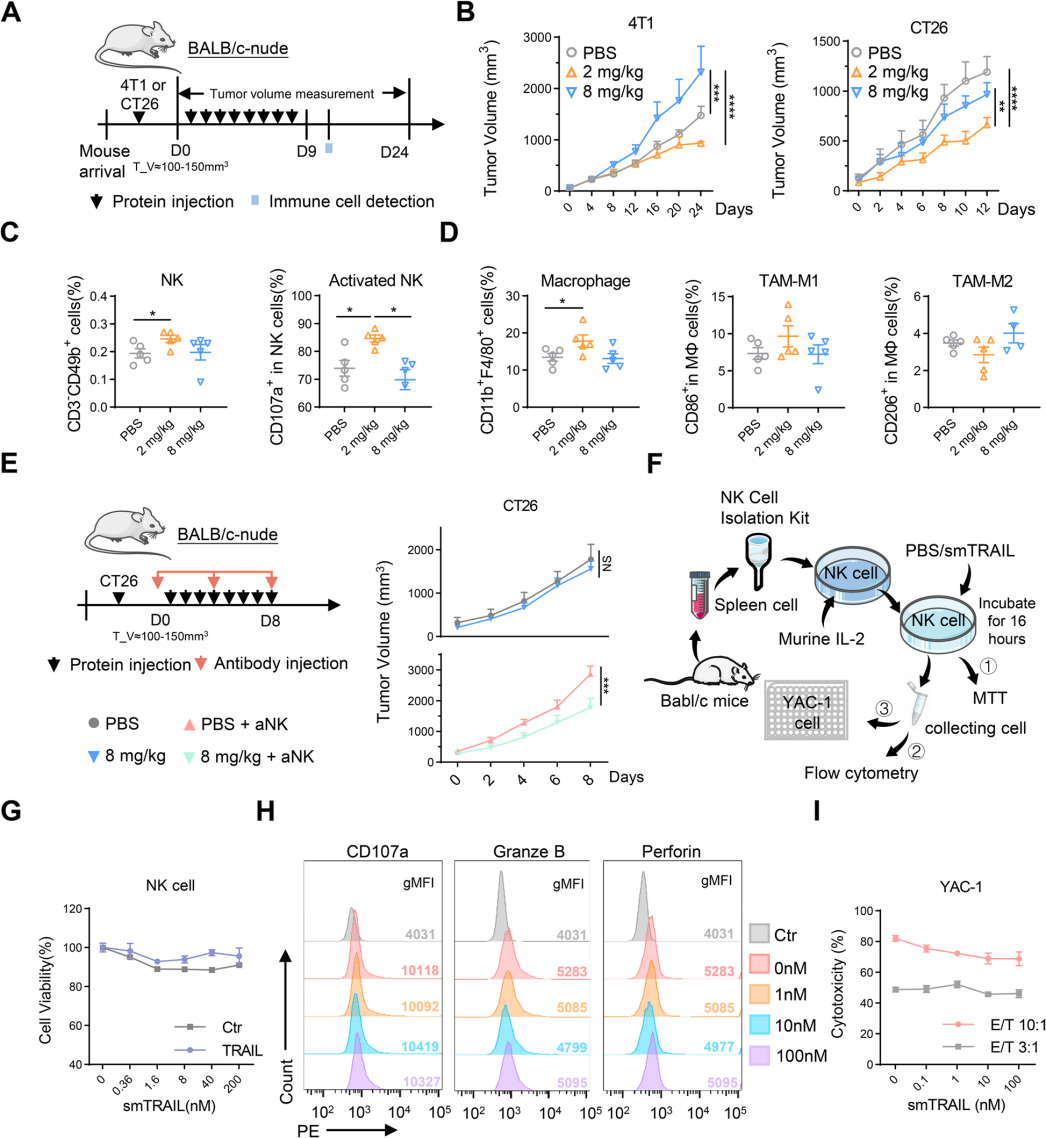
High concentrations of smTRAIL inhibited the number and function of NK cells in BALB/c-Nude mice. **A** Experimental layout. BALB/c-Nude mice were subcutaneously (s.c.) inoculated with 5 × 10^4^ 4T1 cells or 2 × 10^5^ CT26 cells in the right flanks, and smTRAIL was injected intraperitoneally (i.p.) for eight consecutive days (*n* = 6). **B** Tumor growth curves. **C** At 3 d after the last treatment, CT26 tumors were collected and analyzed by flow cytometry to calculate the percentages of intratumoral NK cells (CD3^−^CD49b^+^), activated NK cells (CD3^−^CD49b^+^CD107a^+^) **D** TAMs (CD45^+^F4/80^+^CD11b^+^), M1-TAMs (CD45^+^F4/80^+^CD11b^+^CD86^+^), and M2-TAMs (CD45^+^F4/80^+^CD11b^+^CD206^+^) (*n* = 5). **E** Experimental layout and tumor growth curves in the presence and absence of the NK cell depletion antibody anti-asialo GM1 (*n* = 6). Each mouse was intravenously (i.v.) treated with 20 μL of anti-asialo GM1 every four days. **F** Schematic diagram of the experimental strategy used for the activation of NK cells in vitro. **G** NK cells (5 × 10^4^ per well) were treated with the indicated concentrations of smTRAIL for 16 h, and cell viability was determined by MTT assay. **H** Following incubation of the isolated NK cells with different concentrations of protein for 16 h, the functional marker of NK cells was assayed by flow cytometry. **I** NK cells incubated with the protein were cocultured with YAC-1 cells for 4 h in proportion, and NK cell cytotoxicity was assayed using a lactate dehydrogenase cytotoxicity assay kit. A two-way ANOVA test was used to compare tumor growth responses. One-way ANOVA was performed to calculate the significant differences between groups, followed by LSD analysis. *P < 0.05; ***P < 0.001; and ****P < 0.0001

In 4T1 and CT26 immunocompetent mouse models, we found that NK cells were regulated by treatment with different concentrations of smTRAIL. In addition, smTRAIL treatment reduced the percentage of intratumoral NK cells compared with PBS treatment, especially in the 4T1 model. However, these results have not been previously reported. To explore how this regulatory effect is directly or indirectly caused by smTRAIL, we selected the sensitive cell line 4T1 and insensitive cell line CT26 to establish tumor models in BALB/c-Nude mice, excluding the influence of adaptive immunity. The experimental strategy is illustrated in Fig. 3A. The results of the tumor growth curve showed that treatment with 2 mg/kg smTRAIL inhibited tumor growth to a certain extent in both models, whereas treatment with 8 mg/kg smTRAIL did not affect the growth of CT26 cells and promoted tumor growth in the 4T1 model (Fig. 3B). The tumor suppressor effect in the different tumor models in BALB/c-Nude mice was different from that in immunocompetent mice (Fig. 2B), indicating that the immune regulation of smTRAIL is also related to other immune cells, and that smTRAIL can affect the tumor suppressor effect by affecting innate immune cells in the TME. Since CT26 cells are insensitive to smTRAIL, the clear inhibition of tumor growth in the 2 mg/kg smTRAIL group may be mainly due to the activation of NK cells by the protein. Immune cells were assayed in the tumor tissues of the CT26 model by flow cytometry, and we found that the number of NK cells and the percentage of activated NK cells were significantly increased in the 2 mg/kg smTRAIL group, but not in the 8 mg/kg smTRAIL group, compared to the PBS group (Fig. 3C). A similar pattern was observed for the number of DCs (data not shown). Compared with PBS, the 2 mg/kg smTRAIL group had increased numbers of M1-TAMs, while the 8 mg/kg smTRAIL group had increased numbers of M2-TAMs, although no significant changes were observed after smTRAIL treatment (Fig. 3D). To further verify the role of NK cells, we injected anti-asialo GM1 to deplete NK cells through the tail vein on days 0, 4, and 8 in CT26 tumor-bearing mice (Fig. 3E). We found that treatment with 8 mg/kg smTRAIL effectively inhibited tumor growth in the absence of NK cells, unlike that observed in the presence of NK cells (Fig. 3E). This result indicated that the inhibition of NK cell function by 8 mg/kg smTRAIL counteracted its tumor-killing effect.

To verify whether different concentrations of smTRAIL protein have a direct effect on NK cells, we isolated primary NK cells from the spleens of mice by magnetic bead separation and assayed NK cell activity and function in vitro (Fig. 3F). NK cells activated by IL-2 were incubated with different concentrations of smTRAIL, and the MTT assay showed that smTRAIL did not kill NK cells (Fig. 3G). Flow cytometric analysis showed that smTRAIL did not affect the expression of functional markers in NK cells (Fig. 3H). To determine whether the function of NK cells is affected by smTRAIL, we incubated NK cells after incubation with different concentrations of smTRAIL with the target cell YAC-1 in different proportions, and we tested the NK cell killing activity using a lactate dehydrogenase kit 4 h later. The results showed that smTRAIL did not directly affect NK cell function in vitro (Fig. 3I). These data suggest that different concentrations of smTRAIL dynamically affect the activation and function of NK cells, which are not directly stimulated by smTRAIL.

### smTRAIL treatment induces tumor cells to secrete CCL2 through the TRAIL-TRAIL-R axis

**Fig. 4:**
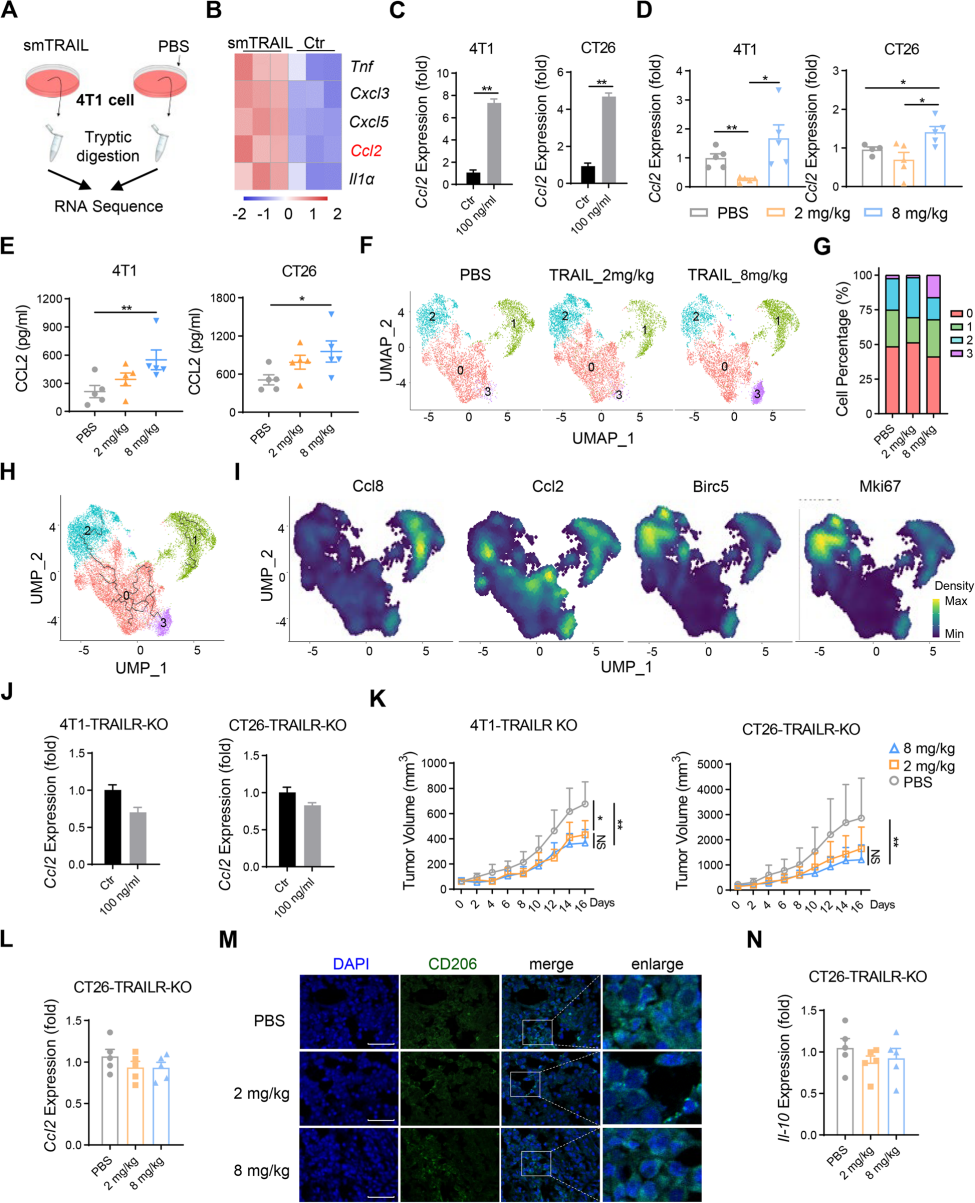
The TRAIL-TRAILR axis leads to CCL2 secretion by tumor cells. **A** Schematic outline of the RNA-seq. After incubation of 4T1 cells with 100 ng/mL smTRAIL or PBS for 24 h, the cells were harvested for transcriptome sequencing. **B** Heatmap of functional genes differentially expressed in smTRAIL and control. **C** After 4T1 cells and CT26 cells were incubated with smTRAIL for 24 h, the levels of *Ccl2* mRNA were analyzed by real-time RT-PCR and normalized to β-actin (*n* = 3). **D** The levels of *Ccl2* mRNA in 4T1 tumor tissue and CT26 tumor tissue was analyzed by real-time RT-PCR and normalized to β-actin (*n* = 5). **E** The secretion of CCL2 in 4T1 tumor tissue and CT26 tumor tissue were analyzed (*n* = 5). **F** UMAP plots demonstrating the cancer cell cluster distribution for each treatment group. **G** Stacked histograms of the frequencies of clusters in the groups. **H** Trajectory plot of macrophage clusters. **I** The expression levels of relative genes are shown in the density plot. **J** After 4T1-TRAILR-KO cells and CT26-TRAILR-KO cells were incubated with smTRAIL for 24 h, the levels of *Ccl2* mRNA were analyzed by real-time RT-PCR and normalized to β-actin (*n* = 3). **K** Tumor growth curves (*n* = 6). **L** The level of *Ccl2* mRNA in CT26-TRAILR-KO tumor tissue was analyzed by real-time RT-PCR and normalized to β-actin (*n* = 5). **M** Representative images of CT26-TRAILR-KO tumor-bearing mice treated with smTRAIL. Tumor tissues stained with DAPI and anti-CD206. Scale bars, 50 μm. **N** The level of *Il-10* mRNA in CT26-TRAILR-KO tumor tissue was analyzed by real-time RT-PCR and normalized to β-actin (*n* = 5). Statistical analysis was carried out by unpaired t-test, one-way ANOVA, and two-way ANOVA test. *P < 0.05; ***P < 0.001; and ****P < 0.0001

Our results showed that treatment with 8 mg/kg of smTRAIL resulted in the inhibition of activated NK and CD8^+^ T cells, and this effect was not directly induced by smTRAIL. Endogenous TRAIL/TRAIL-R-mediated CCL2 secretion with recruitment of M2-like immune cells to tumors via CCR2 has been demonstrated [19]. Therefore, we hypothesized that the smTRAIL-induced cancer secretome promotes a tumor-supportive immune microenvironment. We performed bulk RNA sequencing on 4T1 breast cancer cells treated with smTRAIL (Fig. 4A). This analysis revealed the efficient activation of the chemokine signaling pathway (Fig. S4A) and high expression of the chemokine genes *Cxcl3*, *Cxcl5*, and *Ccl2* (Fig. 4B). Among these, *Cxcl3* and *Cxcl5* are potent neutrophil attractors and activators [34], whereas *Ccl2* is a key chemokine that regulates monocyte/macrophage migration and infiltration [35]. In vitro and in vivo analyses showed that smTRAIL could induce both 4T1 and CT26 cells to express *Ccl2*, and treatment with 8 mg/kg smTRAIL significantly increased the expression of CCL2 in tumors from both models (Fig. 4C, D, and E). ScRNA-seq analysis of tumor cells after smTRAIL treatment in CT26 tumor-bearing models was also performed. We investigated alterations in tumor cell subtypes among the smTRAIL-treated and control groups and found that relatively independent subtypes (cluster 3) were predominantly present in the 8 mg/kg smTRAIL-treated group (Fig. 4F and G). RNA trajectory analysis suggested that the trajectory of cluster 3 was associated with that of clusters 0 and 2 (Fig. 4H). Gene expression analysis showed that chemokines such as *Ccl2* and *Ccl8* and cell proliferation markers such as *Mki67* and *Birc5* were highly expressed in cluster 3 (Fig. 4I), indicating that 8 mg/kg of smTRAIL may induce tumor cell “activation” and secretion of chemokines to recruit immune cells. To further verify whether TRAIL-R expression in cancer cells results in CCL2 production by tumor cells and thereby facilitates the accumulation of M2 macrophages in the TME, we knocked out TRAIL-R expression on the surface of tumor cells using CRISPER-Cas9 (Fig. S4B), and found that both 4T1-TRAIL-R-KO and CT26-TRAIL-R-KO cell lines blocked TRAIL-TRAIL-R axis-induced *Ccl2* expression (Fig. 4J). Using TRAIL-R-KO cell lines for tumor formation, both 2 mg/kg smTRAIL and 8 mg/kg smTRAIL treatments significantly inhibited tumor growth in vivo (Fig. 4K). In contrast to the 4T1 and CT26 models, *Ccl2* mRNA expression in CT26-TRAIL-R-KO tumor tissues was not affected by smTRAIL treatment, whereas *Ccl2* mRNA expression in 4T1-TRAIL-R-KO tumor tissues was significantly inhibited in the smTRAIL-treated groups (Fig. S4C and Fig. 4L). We also detected changes in macrophages by isolating CT26-TRAIL-R-KO tumor tissues for immunofluorescence analysis and found no significant difference in the number of infiltrating M2 macrophages between the 2 and 8 mg/kg smTRAIL groups (Fig. 4M). In addition, *IL-10* mRNA expression in both TRAIL-R KO tumor tissues was disrupted in the 8 mg/kg smTRAIL group (Fig. S4D and Fig. 4N). In conclusion, immunosuppression in the TME induced by 8 mg/kg smTRAIL was associated with smTRAIL-induced CCL2 secretion by tumor cells via the TRAIL-TRAILR axis.

### smTRAIL treatment increases the percentage of C1q^+^ TAMs with higher CCR2 expression in TME

**Fig. 5:**
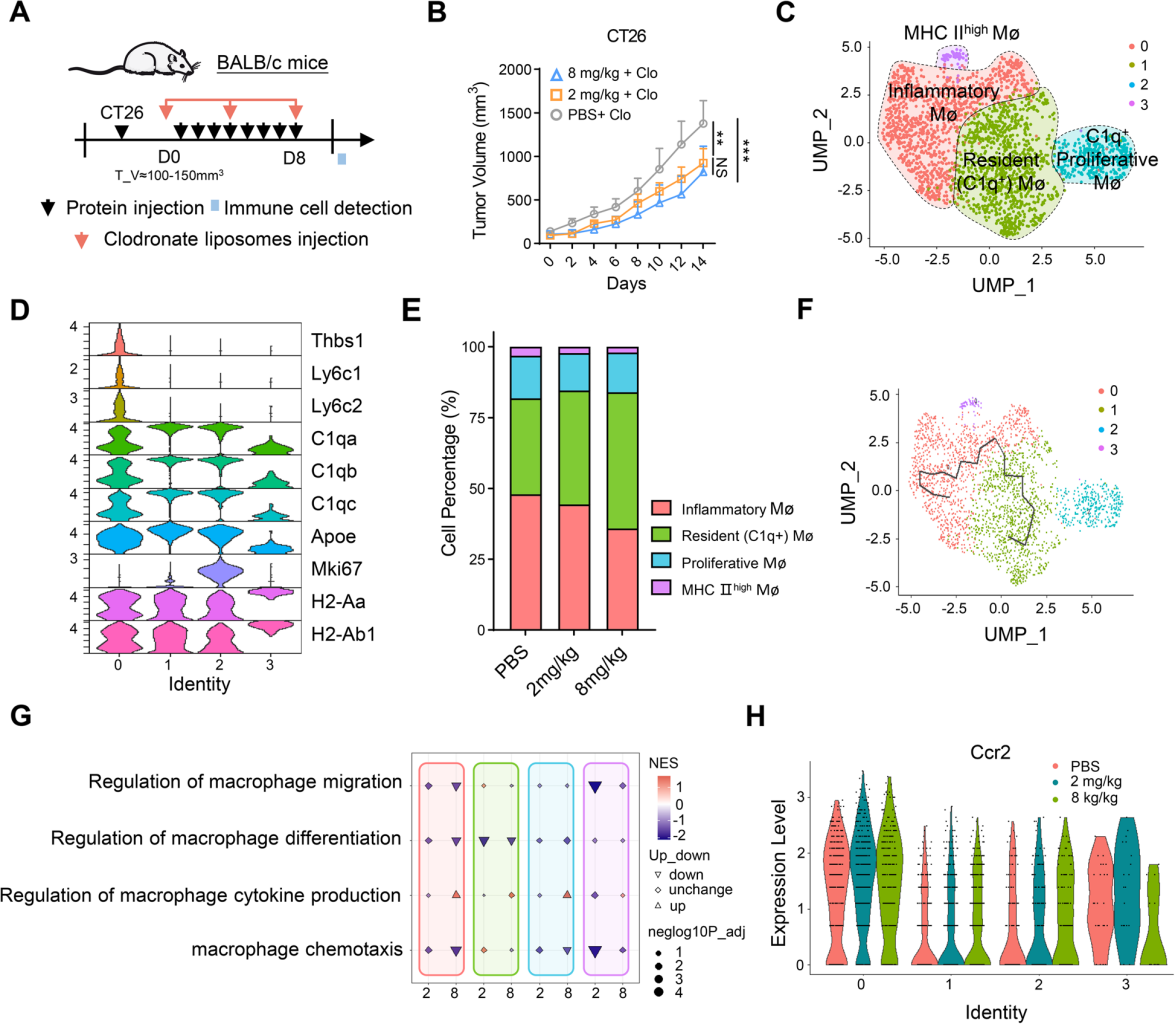
Evaluation of macrophage changes in vivo and exploration of the mechanism. **A** Experimental layout. BALB/c mice were subcutaneously (s.c.) inoculated with 2 × 10^5^ CT26 cells in the right flanks, smTRAIL was injected intraperitoneally (i.p.) for eight consecutive days, and each mouse was intravenously (i.v.) treated with 50 μg/kg clodronate liposomes every four days (*n* = 6). **B** Tumor growth curves. **C** UMAP plot of reclustered macrophage cells from Fig. S4A. **D** Expression of key genes used for identification of cell clusters from (C) by violin plot. **E** Stacked histograms of the frequencies of macrophage cell clusters in the groups. **F** Trajectory plot of macrophage clusters. **G** GOBP analysis performed in all macrophage cells comparing 2 mg/kg versus 8 mg/kg. **H** Violin plot showing the expression of Ccr2 in macrophage cell clusters. A two-way ANOVA test was used to compare the tumor growth responses. **P < 0.01; and ***P < 0.001

Flow cytometric analysis revealed that TAMs were modulated by smTRAIL treatment. To determine whether macrophages are involved in the smTRAIL-mediated antitumor effect, we treated mice with the macrophage scavenger clodronate liposomes in the CT26 model, as described previously (Fig. 5A) [36, 37]. As expected, after macrophages were effectively depleted (Fig. S5A), the tumor-promoting effect was effectively reversed in the 8 mg/kg smTRAIL-treated group (Fig. 5B). We also found that macrophage depletion disrupted the inhibitory effect of 8 mg/kg smTRAIL on NK cell activation (Fig. S5B). To further explore the role of macrophages in the TME, we examined the gene expression patterns of TAMs using scRNA-seq data from the CT26 model. Treatment with 8 mg/kg smTRAIL induced a total of 428 differentially expressed genes (DEGs), and treatment with 2 mg/kg smTRAIL induced 44 DEGs compared to the PBS group (Fig. S5C), indicating that high-dose smTRAIL treatment had a greater influence on the macrophage transcriptome. Macrophages were divided into four subsets based on the expression of specific genes (Figs. 5C and D, Fig. S5D), namely resident (C1q^+^) Mø (*C1qa*, *C1qb*, *C1qc*, and *Apoe*), inflammatory Mø (*Thbs1*, *Ly6c,* and *Plac8*), proliferative Mø (*Mki67*), and MHC class II ^high^ Mø (*H2-Aa and H2-Ab1*) [38, 39]. The results showed that treatment with 8 mg/kg smTRAIL significantly increased the infiltration of C1q^+^ macrophages (clusters 1 and 2) and significantly reduced the number of inflammatory macrophages (Fig. 5E). Cluster trajectory analysis showed that Cluster 1 was related to Cluster 0. Combined with the cluster percentage changes and trajectory analysis, it appears that treatment with 8 mg/kg smTRAIL may induce the conversion of inflammatory Mø (cluster 0) to C1q^+^ Mø cells (cluster 1) (Fig. 5F). TRAIL treatment downregulated biological processes, including macrophage migration/differentiation and chemotaxis, and upregulated cytokine production (Fig. 5G). In addition, we found that smTRAIL treatment increased the expression of *Ccr2* in resident C1q^+^ Mø (cluster 2) (Fig. 5H), which would facilitate the recruitment of C1q^+^ TAMs by CCL2 in the TME. We also found that C1q^+^ macrophages are highly active in pathways that contribute to the regulation of macrophage migration and chemokine-mediated signaling pathways. Spots with inflammatory macrophages were associated with the positive regulation of cell death and negative regulation of cytokine production (Fig. S5E). These results showed that smTRAIL treatment increased the percentage of C1q^+^ TAMs with higher CCR2 expression in the TME and that the TAMs affected the antitumor effects in the CT26 tumor model. Recent studies have suggested that complement C1q is a marker of tolerogenic and immunosuppressive macrophage populations in both healthy and tumor tissues, and C1q^+^ TAMs can drive cancer progression by favoring T-cell exhaustion and M2-TAM polarization [40]. Therefore, the promotion of C1q^+^ TAMs in tumors may be correlated with the inhibition of tumor-suppressive immune cell activation induced by treatment with 8 mg/kg smTRAIL.

### smTRAIL directly induces M2-like macrophage polarization through upregulating FoxO1 expression

**Fig. 6:**
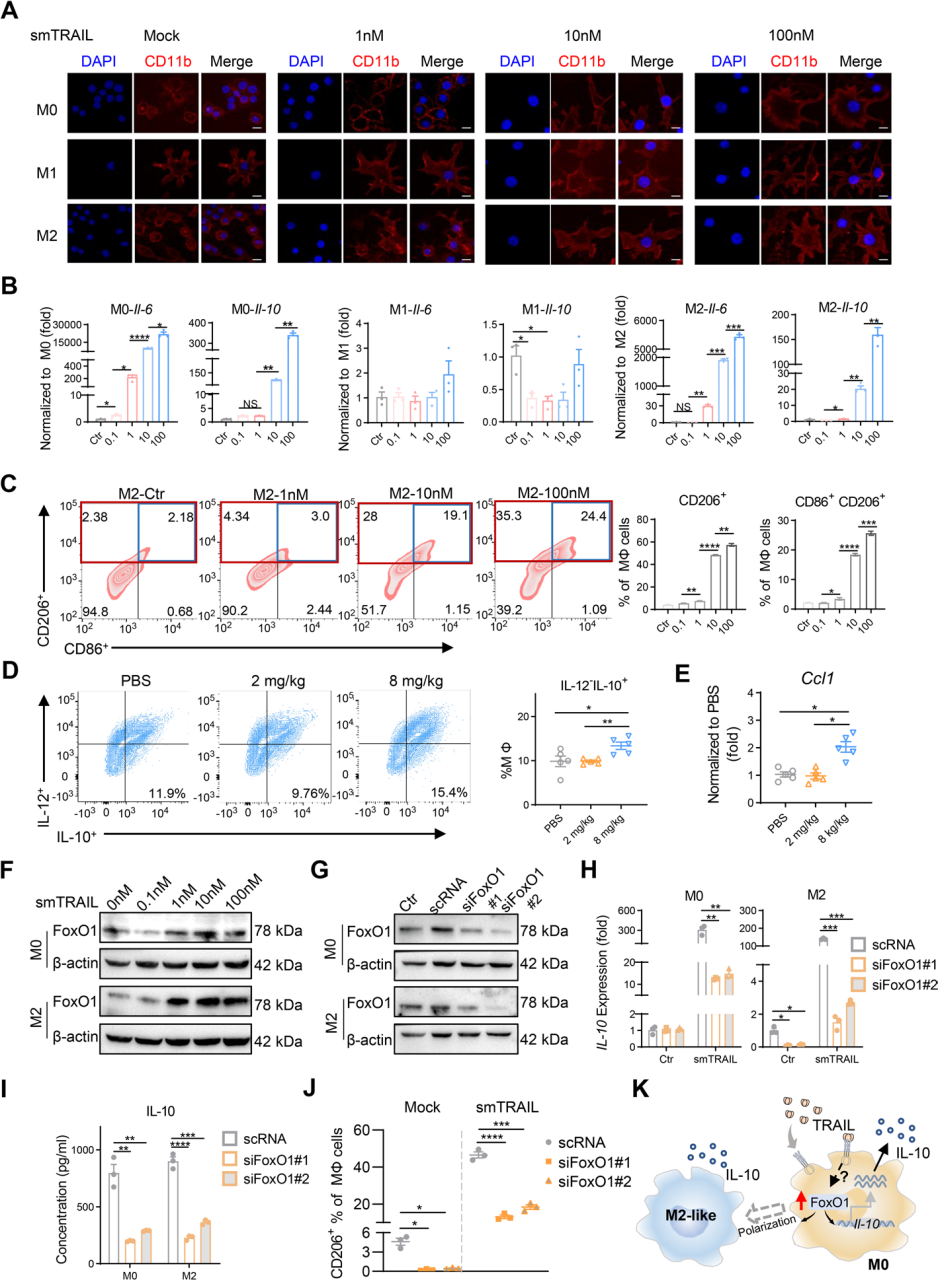
smTRAIL exerts direct effects on macrophages in vitro. **A** M0, M1, and M2-like macrophage cell morphologies by immunofluorescence microscopy. Different concentrations of protein were incubated with macrophages for 24 h, and the cell morphology was determined by CD11b and DAPI staining. Scale bar, 10 μm. **B** The levels of *Il-6* mRNA and *Il-10* mRNA in M0, M1, and M2-like macrophages after smTRAIL incubation analyzed by real-time RT-PCR and normalized to β-actin (*n* = 3). **C** The percentages of CD206^+^ M2-like macrophages (red frame) and CD86^+^CD206^+^ M2-like macrophages (blue frame) were detected by flow cytometry. **D** The percentage of M2b-like macrophages (IL-12^−^ IL-10^+^ macrophages) in CT26 tumor tissues was detected by flow cytometry (*n* = 5). **E** Levels of mRNA expression of *Ccl1* in CT26 were analyzed by real-time RT-PCR and normalized to β-actin (*n* = 5). **F** FoxO1 expression in macrophages by western blot. After incubating M0 and M2-like macrophages for 24 h with different protein levels, the cells were harvested, and the expression of FoxO1 was detected by SDS-PAGE followed by western blotting with (C29H4) rabbit mAb against FoxO1. **G** Western blot of FoxO1 expression after silencing of the FoxO1 gene in macrophages. **H** The level of *Il-10* mRNA was analyzed by real-time RT-PCR and normalized to β-actin after silencing of the FoxO1 gene in M0 and M2-like macrophages (*n* = 3). **I** IL-10 expression analyzed by ELISA after silencing of the FoxO1 gene in M0 and M2-like macrophages (*n* = 3). **J** After FoxO1 silencing, M2-like macrophages were incubated with and without smTRAIL for 24 h. The percentage of CD206^+^ M2-like macrophages was determined by flow cytometry. **K** Mechanism of smTRAIL acting on macrophages. A one-way ANOVA test was performed to calculate the significant differences between groups, followed by LSD analysis. *P < 0.05; **P < 0.01; ***P < 0.001; and ****P < 0.0001

Our scRNA-seq analysis suggests that there may be phenotypic conversions in different types of macrophages following smTRAIL treatment. Previous studies have reported that TRAIL directly induces tumor-supportive immune cell apoptosis [14, 17, 41]. Therefore, we explored whether smTRAIL directly affected TAM polarization and phenotypic conversion. We obtained M1-like macrophages by inducing mouse RAW264.7 macrophages with LPS, and M2-like macrophages were induced by IL-4 and IL-10 [42, 43]. The phenotypes were confirmed by typing-related mRNA and cell marker detection (Figs. S6A to D). We incubated M0, M1, and M2 macrophages with different concentrations of smTRAIL for 24 h and found that with an increase in smTRAIL concentration, the morphology of M0 and M2 macrophages changed, becoming similar to that of the M1 phenotype with a radial shape (Fig. 6A and Fig. S6E). However, smTRAIL had no obvious effect on the morphology of the M1 macrophages. To determine whether M0 and M2 macrophages became polarized to M1 macrophages, we assayed the expression of M1-related genes and found that these genes increased with increasing protein concentration in both macrophage types (Figs. S7A and B). However, we also found that the levels of the M2-related genes *Il-10* and *Il-6* were also increased with increasing protein concentration, whereas that of M1 was not affected by the protein (Fig. 6B). To explore its polarization state, surface markers were detected by flow cytometry, which showed that the percentage of CD206^+^ macrophages in M0 and M2 increased, and the percentages increased with increased protein concentrations (Fig. S7C and Fig. 6C). The proportion of M2b cells (CD86^+^CD206^+^ macrophages), a subtype of M2 macrophages, also exhibited dose-dependent effects. In addition, the percentages of IL-10^+^ M2 and IL-10^+^ M2b macrophages were dose-dependent, as IL-10 is the primary marker of M2b macrophages (Figs. S7D and E). These findings suggest that smTRAIL directly promotes M2-like macrophage polarization in vitro. M2b macrophages are formed under the action of immune complexes or TLR agonists, mainly secrete cytokines such as IL-10, IL-6, and TNF, and perform immunomodulatory functions [44]. We also examined the proportion of M2b macrophages in CT26 tumor tissues and found that treatment with 8 mg/kg smTRAIL significantly increased M2b macrophage infiltration (high IL-10 and low IL-12 levels) (Fig. 6D). The mRNA level of the M2b-specific marker, CCL1, was also significantly increased in tumors in the 8 mg/kg smTRAIL group (Fig. 6E). These results suggest that smTRAIL induces macrophage polarization towards M2-like macrophages in the TME.

Next, we explored the molecular mechanism of the M2-like macrophage polarization induced by smTRAIL. Several factors, such as posttranscriptional regulators, signaling molecules, transcription factors, and physical factors, have been found to play pivotal roles in controlling M2 macrophage polarization [45]. Chung et al. previously found that FoxO1, a member of the FoxO family of transcription factors, enhances IL-10 expression by directly binding to the IL-10 promoter, and contributes to the polarization of M2 macrophages [46, 47]. Therefore, we determined whether smTRAIL affects the expression of FoxO1 and contributes to macrophage polarization towards the M2-like phenotype by enhancing IL-10 expression. As expected, we found that the expression of FoxO1 was dependent on the dose of smTRAIL (Fig. 6F), and that the *FoxO1* mRNA expression level was consistent with the protein level results in M2, although there was no significant difference in M0 (Fig. S7F). These data suggest that smTRAIL enhances the expression of FoxO1 at the transcriptional level in TAMs. To verify the distinct role of FoxO1, we silenced FoxO1 expression using siRNA (Fig. 6G and Fig. S7G). Compared to no silencing, FoxO1 silencing significantly inhibited the mRNA expression of *Il-10* and the expression of IL-10 in the supernatant (Fig. 6H and I). In addition, macrophage marker expression analysis showed that FoxO1 silencing significantly reduced the expression of CD206 in the presence or absence of smTRAIL (Fig. 6J). The mechanism of smTRAIL-induced M2-like macrophage polarization is shown in Fig. 6K. Taken together, these results suggest that a certain concentration of smTRAIL can directly modulate M2-like macrophage polarization by upregulating the expression of FoxO1 in TAMs.

### Immunoregulatory effects of shTRAIL in a humanized immune system mouse model

**Fig. 7:**
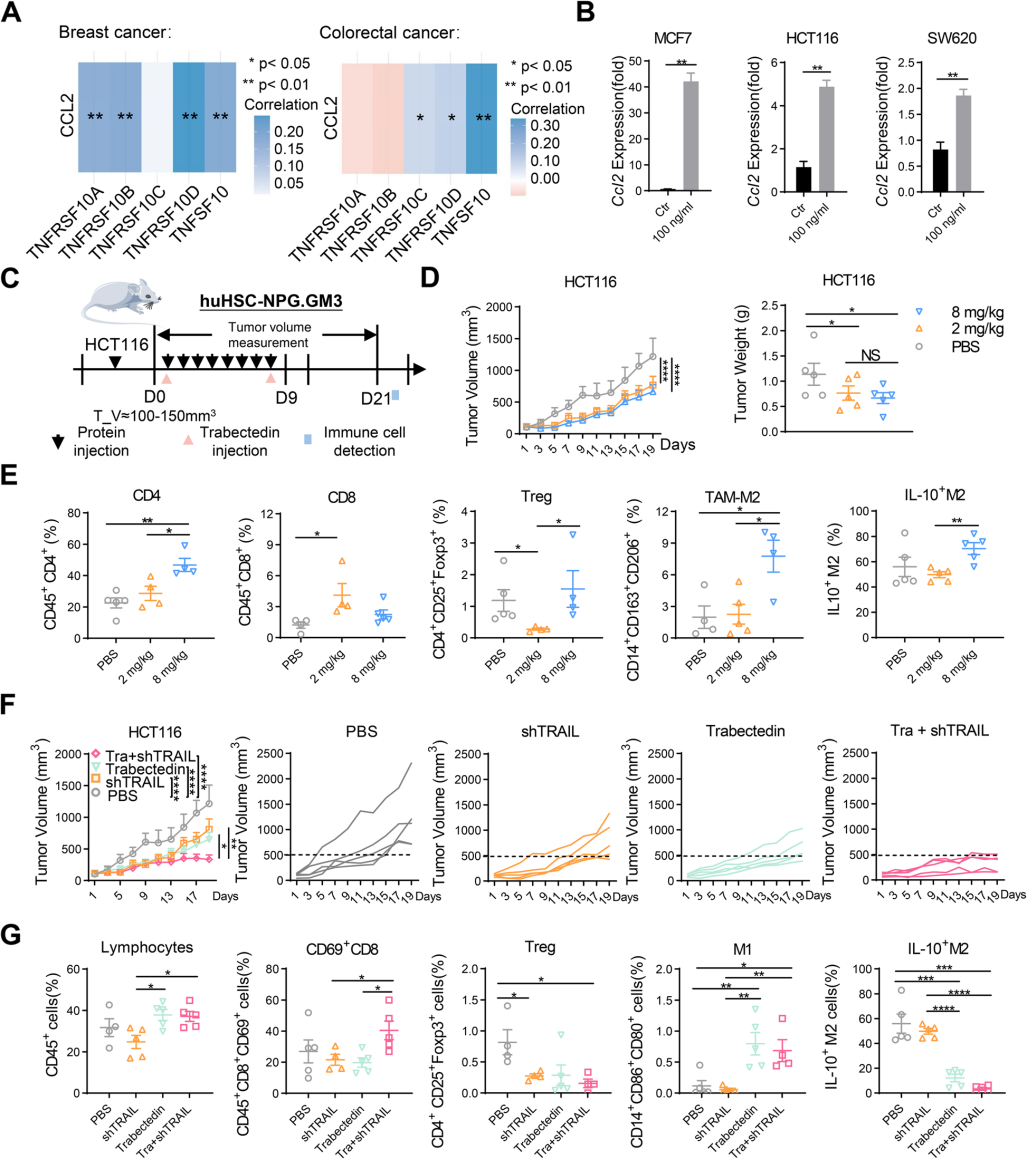
Immunomodulatory function of shTRAIL and combination therapy in humanized mice. **A** Correlation analysis of CCL2 with TRAIL or TRAIL-R in breast and colorectal cancer based on the TCGA database (TNFRSF10A, TNFRSF10B, TNFRSF10C, and TNFRSF10D were TRAIL-R; TNFSF10 was TRAIL). **B** After MCF7, HCT116, and SW620 cells were incubated with shTRAIL for 24 h, the levels of *Ccl2* mRNA were analyzed by real-time RT-PCR and normalized to GAPDH (*n* = 3). **C** Experimental layout. huHSC-NPG.GM3 mice were subcutaneously (s.c.) inoculated with 1 × 10^6^ HCT116 cells in the right flanks, and shTRAIL was injected intraperitoneally(i.p.) for eight consecutive days. At 21 d, the immune cells were assayed by flow cytometry (*n* = 5). **D** Tumor growth curves and tumor weights. **E** The percentages of intratumoral CD4^+^ T cells (CD45^+^CD4^+^), CD8^+^ T cells (CD45^+^CD8^+^), Tregs (CD45^+^CD4^+^CD25^+^Foxp3^+^), M2-TAMs (CD14^+^CD163^+^CD206^+^), and IL-10^+^ M2 (*n* = 5). **F** Tumor growth in humanized mice. Mice bearing HCT116 tumors were treated with PBS, shTRAIL (2 mg/kg), trabectedin (0.15 mg/kg/per mouse), or a combination of shTRAIL and trabectedin. Trabectedin was administered intravenously (i.v.) once a week. **G** The percentages of intratumoral lymphocytes (CD45^+^), activated CD8^+^ T cells (CD45^+^CD8^+^CD69^+^), Tregs (CD45^+^CD4^+^CD25^+^Foxp3^+^), M1-TAMs (CD14^+^CD86^+^CD80^+^), and IL-10^+^ M2(CD14^+^CD163^+^CD206^+^IL-10^+^) (*n* = 5). A two-way ANOVA test was used to compare the tumor growth responses. One-way ANOVA was performed to calculate the significant differences between groups, followed by LSD analysis. *P < 0.05; **P < 0.01; ***P < 0.001; and ****P < 0.0001

Given that smTRAIL treatment exerts different immunoregulatory effects at different doses in immunocompetent mice, we further explored whether these immunoregulatory effects are responsible for soluble human TRAIL (shTRAIL) treatment outcomes in human tumors. Analysis of the TCGA database showed that the expression of CCL2 was significantly higher in breast cancer samples than in normal tissues, whereas the opposite was true in colorectal cancer samples (Fig. S8A). However, CCL2 expression was significantly associated with human TRAIL and TRAIL-R expression in both cancer types (Fig. 7A). Different concentrations of shTRAIL were incubated with breast MCF7, colorectal HCT116, and SW620 cancer cells. MTT assays showed that the protein effectively killed tumor cells (Fig. S8B). This protein significantly increased *Ccl2* mRNA levels after incubation with tumor cells (Fig. 7B). To explore the immunomodulatory effects of shTRAIL in the human immune microenvironment, we generated HU-HSC-NPG.GM3 humanized immune system mouse model with detectable expression of human macrophages and NK cells in HCT116 tumor-bearing cells. Figure 7C shows a schematic diagram of the treatment and detection. Treatment of mice with 2 and 8 mg/kg shTRAIL resulted in a significant reduction in tumor growth, but there was no significant difference between these two doses (Fig. 7D). Analysis of immune cells showed that treatment with 2 mg/kg of shTRAIL significantly increased the number of CD8^+^ T cells and reduced the number of Tregs compared to that in the PBS group. Compared to the 2 mg/kg protein treatment group, 8 mg/kg shTRAIL treatment not only significantly increased CD4^+^ T cells, Tregs, and M2 macrophages in the tumor but also upregulated IL-10 expression in intratumoral M2 macrophages (Fig. 7E). These data suggest that shTRAIL plays an immunoregulatory role in the human TME and indicate that relatively high-dose protein treatment also has an immunosuppressive effect by increasing M2-like macrophages in the tumor.

### Combination of shTRAIL with trabectedin remodels the TME and results in improved antitumor effects in a tumor-bearing humanized mouse model

Although 2 mg/kg recombinant TRAIL treatment showed an antitumor immune effect, immunosuppressive cells and cytokines, such as M2 macrophages and CCL2, may still have limited antitumor effects. Therefore, we investigated whether inhibition of CCL2 or M2 macrophages enhances the antitumor immunity of shTRAIL in tumor-bearing humanized mice. We tested the chemotherapeutic drug trabectedin, which has been reported to inhibit CCL2 secretion and selectively deplete monocytes/macrophages in tumors [18, 48], in combination with 2 mg/kg of shTRAIL in HCT116-bearing HU-HSC-NPG.GM3 humanized mice (Fig. 7C). Compared with the PBS group, all three treatment groups (shTRAIL, trabectedin, and their combination) significantly inhibited tumor growth. Tumor inhibition rates were 33.7%, 46.6%, and 72.7%, respectively. The antitumor efficiency of the combination treatment group was 2.2 times that of the shTRAIL treatment group (Fig. 7F). From the immune cell assay, it was found that trabectedin can upregulate intratumoral CD45^+^ immune cells and M1 macrophages in tumors, and it reduced the number of immunosuppressive cells, including Tregs and IL-10^+^ M2-like macrophages. The combination treatment not only significantly increased the number of activated CD8^+^ T cells, but also significantly inhibited the proliferation of Treg cells and IL-10^+^ M2 macrophages (Fig. 7G). Together, these results suggest that the combination of shTRAIL with a CCL2 inhibitor results in improved antitumor effects by remodeling the tumor immune microenvironment, thus demonstrating the therapeutic benefit of combining shTRAIL with the chemotherapeutic trabectedin and underscoring the potential of this approach for clinical translation.

## Discussion

Conflicting effects of TRAIL on immunity have been reported and have led to its use as a double-edged sword. Nevertheless, the immunomodulatory mechanisms and role of recombinant TRAIL in the tumor immune microenvironment are unclear. In this study, we investigated the immunoregulatory effects of exogenous recombinant TRAIL in the TME using soluble murine TRAIL analyzed in immunocompetent mice and validated soluble human TRAIL in a humanized immune system mouse model. Interestingly, the dose-related bidirectional regulatory role of recombinant TRAIL in the TME was revealed for the first time in our study. We found that an optimal dose of smTRAIL (2 mg/kg) could effectively activate DCs, NK cells, CD4^+^ and cytotoxic CD8^+^ T cells in the TME of both apoptosis-sensitive and apoptosis-insensitive cell lines, thus enhancing its antitumor effect. However, we also found that a higher dose of smTRAIL (8 mg/kg) could induce immunosuppression to inhibit both NK cell and CD8^+^ T-cell activation, which counteracts the tumor-killing effect caused by the induction of tumor apoptosis and even promotes the tumor growth of insensitive cells. Considering that 8 mg/kg recombinant TRAIL is only an intermediate dose in clinical studies (0.5–30 mg/kg) [6], we emphatically determined the mechanism of its immunosuppressive effect.

Analysis of intratumoral immune cells by flow cytometry and scRNA-seq revealed that TAMs play an important role in immunosuppression triggered by treatment with 8 mg/kg smTRAIL. Specifically, treatment with a higher dose of smTRAIL allowed tumor cells to recruit TAMs (mainly C1q^+^ macrophages) by secreting CCL2. smTRAIL also promotes M2-like macrophage polarization and increases the production of pro-inflammatory cytokines, such as IL-10 and VEGF, which inhibit the activation of NK cells and CD8^+^ T cells. This mechanism supports the findings of Hartwig et al., who reported that the TRAIL-induced cancer secretome promotes a tumor-supportive immune microenvironment via CCR2 in apoptosis-resistant cancer cells [19]. Therefore, in our study, we found that a higher dose of smTRAIL elicited immunosuppression in both apoptosis-sensitive and apoptosis-insensitive cell lines. Moreover, we further investigated and confirmed the key role of TAMs in the immunomodulatory effects of recombinant TRAIL and found an interaction between smTRAIL and TAMs.

TAMs display remarkable plasticity within the TME and can switch from one phenotype to another [49]. The opposing effects of antitumor M1-like and protumor M2-like cells on tumors directly affect the current strategies aimed at improving antitumor immune responses [50]. In this study, we also found that smTRAIL could directly affect M2-like macrophage polarization. M2 macrophages can be further subdivided into M2a, M2b, M2c, and M2d based on the applied stimuli and the resultant transcriptional changes [51]. As an M2 subtype, M2b macrophages have potent immunomodulatory and anti-inflammatory effects that promote tumor progression by blunting immune and inflammatory responses [45]. A wide variety of stimuli and factors modulate M2b macrophage polarization in vitro and in vivo. Furthermore, Kobayashi et al. reported that treatment with recombinant CCL2 could induce M2b polarization in resident monocytes [52]. This indicates that CCL2 secreted by tumor cells after treatment with 8 mg/kg of smTRAIL may also contribute to M2b polarization in vivo. Notably, we found that recombinant TRAIL directly induced M2-like TAM polarization in vitro. We also showed that the transcription factor FoxO1 is likely involved in the upregulation of IL-10 and M2-like macrophage polarization induced by smTRAIL. Recent studies have shown that FoxO1 stimulates the expression of proinflammatory IL-1β and enhances TLR4 signaling in mature macrophages [53], supporting our data since IL-1β is also used to induce macrophage polarization to M2b in vitro [54]. In addition, our results also suggest that C1q^+^ TAMs may be the origin of M2-like macrophages, since the scRNA-seq analysis showed that C1q^+^ TAMs significantly increased in the 8 mg/kg smTRAIL treatment group. These results support recent findings and the hypothesis that C1q^+^ TAMs can promote protumoral M2-like macrophage polarization in cancers [35, 55].

Despite encouraging preclinical results with recombinant TRAIL, it failed to show any significant anticancer activity in clinical trials. Our results will also help guide the clinical administration and therapeutic strategies of recombinant TRAIL in tumor treatment. First, an optimal therapeutic dose may need to be evaluated for different patients and tumor types based on immune responses. Notably, we showed that at the same dose of smTRAIL (8 mg/kg), insensitive tumor CT26 cells secreted more CCL2 and recruited more macrophages than sensitive 4T1 cells, which may support tumor growth. This indicates that it is complex to adjust the dose of recombinant TRAIL for clinical treatment. Second, recombinant TRAIL can be combined with immunotherapy for the treatment of some cancers, including those that are insensitive to apoptosis. Third, optimization strategies for recombinant TRAIL should be considered to avoid immunosuppression and improve antitumor immunity. (i) Systemic delivery using nanoparticles to achieve controlled release or delivery using viral vectors. Recently, we found that the oncolytic adenovirus-mediated intratumoral expression of hTRAIL induces an increase or activation of tumor-infiltrating T cells [56]. These studies suggest that the viral delivery of soluble TRAIL could be a highly effective strategy for success in clinical practice. (ii) Combination therapy to suppress M2-like macrophages or CCL2. We showed that the combination of a macrophage-targeting drug, trabectedin, and an optimal dose of shTRAIL resulted in outstanding antitumor effects in a tumor-bearing humanized mouse model. Moreover, combining chemotherapeutic drugs to inhibit M2-like macrophage polarization or to convert them to M1 macrophages is also a proposed therapeutic concept.

In this study, we found that different doses of recombinant sTRAIL treatment had different immunomodulatory functions, leading to different therapeutic effects. Whether different forms of TRAIL will produce similar results still needs to be verified. In follow-up studies we will express full-length TRAIL using adenoviral vectors to verify its immunomodulatory function in vivo. In addition, we observed a direct effect of smTRAIL on M2-like macrophage polarization, yet further studies are also needed to investigate the precise molecular mechanisms in vitro and in vivo. Our ongoing research has found that lncRNA-MM2P, which was identified as a modulator of macrophage M2 polarization [57, 58] was highly expressed in the immune microenvironment after smTRAIL treatment (data not shown). Therefore, in the future, we will further explore the related molecular mechanisms through RNA sequencing, lncRNA microarrays and gene knockout mouse models, etc.

## Conclusion

This study documented the immunomodulatory role of recombinant TRAIL in the TME and revealed the mechanism of the immunosuppressive effect triggered by higher doses of smTRAIL in different tumor models. Notably, our data highlight the therapeutic potential of combining recombinant TRAIL with trabectedin to remodel the tumor immune microenvironment, which can maximize the antitumor effect of recombinant TRAIL.

### Supplementary Information

**Additional file 1.**

## Data Availability

All data associated with this study are presented in the paper or in the Supplementary Materials. The antibodies, reagents, and software used are listed in Supplementary Table S1. Single-cell RNA sequencing data can be downloaded from public databases (BioProject ID: PRJNA1003149). Further information and requests for resources and reagents should be directed to and will be fulfilled by the lead contact Dr. Bin Yu (yubin@jlu.edu.cn).

## Abbreviations

TRAIL: Tumor necrosis factor (TNF)-related apoptosis-inducing ligand
smTRAIL: Soluble murine TRAIL
shTRAIL: Soluble human TRAIL
TRAs: TRAIL receptor agonists
NK cell: Natural killer cell
DCs: Dendritic cells
CTL: Cytotoxic T lymphocyte
TME: Tumor microenvironment
MDSCs: Myeloid-derived suppressor cells
Tregs: Regulatory T cells
TCGA: The Cancer Genome Atlas
GEO: Gene Expression Omnibus
CAFs: Carcinoma-associated fibroblasts
TCR: T-cell receptor
DEGs: Differentially expressed genes
CIBERSORT: Cell-type identification by estimating relative subsets of RNA transcripts
DMEM: Dulbecco’s modified Eagle’s medium
FBS: Fetal bovine serum
GO: Gene Ontology
KEGG: Kyoto Encyclopedia of Genes and Genomes
CCL2: Chemokine (C–C motif) ligand 2
CCR2: Chemokine (C–C motif) receptor 2
FoxO1: Forkhead box O1
