# Supplementary material for: Dose-related immunomodulatory effects of recombinant TRAIL in the tumor immune microenvironment
Source: J Exp Clin Cancer Res. 2023 Aug 22;42:216. doi: 10.1186/s13046-023-02795-x (PMC10464183; doi:10.1186/s13046-023-02795-x)
Supplement: Supplementary file 1 — Additional file 1. [file 13046_2023_2795_MOESM1_ESM.docx]

**SUPPLEMENTAL INFORMATION**

**Dose-related immunomodulatory effects of recombinant TRAIL in the tumor immune microenvironment**

Xupu Wang^1^, Lizheng Wang^1, 3^, Wenmo Liu^1^, Xinyao Liu^1^, Xinyuan Jia^1^, Xinyao Feng^1^, Fangshen Li^1^, Rui Zhu^1^, Jiahao Yu^1^, Haihong Zhang^1^, Hui Wu^1^, Jiaxin Wu^1^, Chu Wang^1^, Bin Yu^1^* and Xianghui Yu^1,2^*

Corresponding author: Xianghui Yu, [xianghui@jlu.edu.cn](mailto:xianghui@jlu.edu.cn)

Bin Yu, [yubin@jlu.edu.cn](mailto:yubin@jlu.edu.cn)


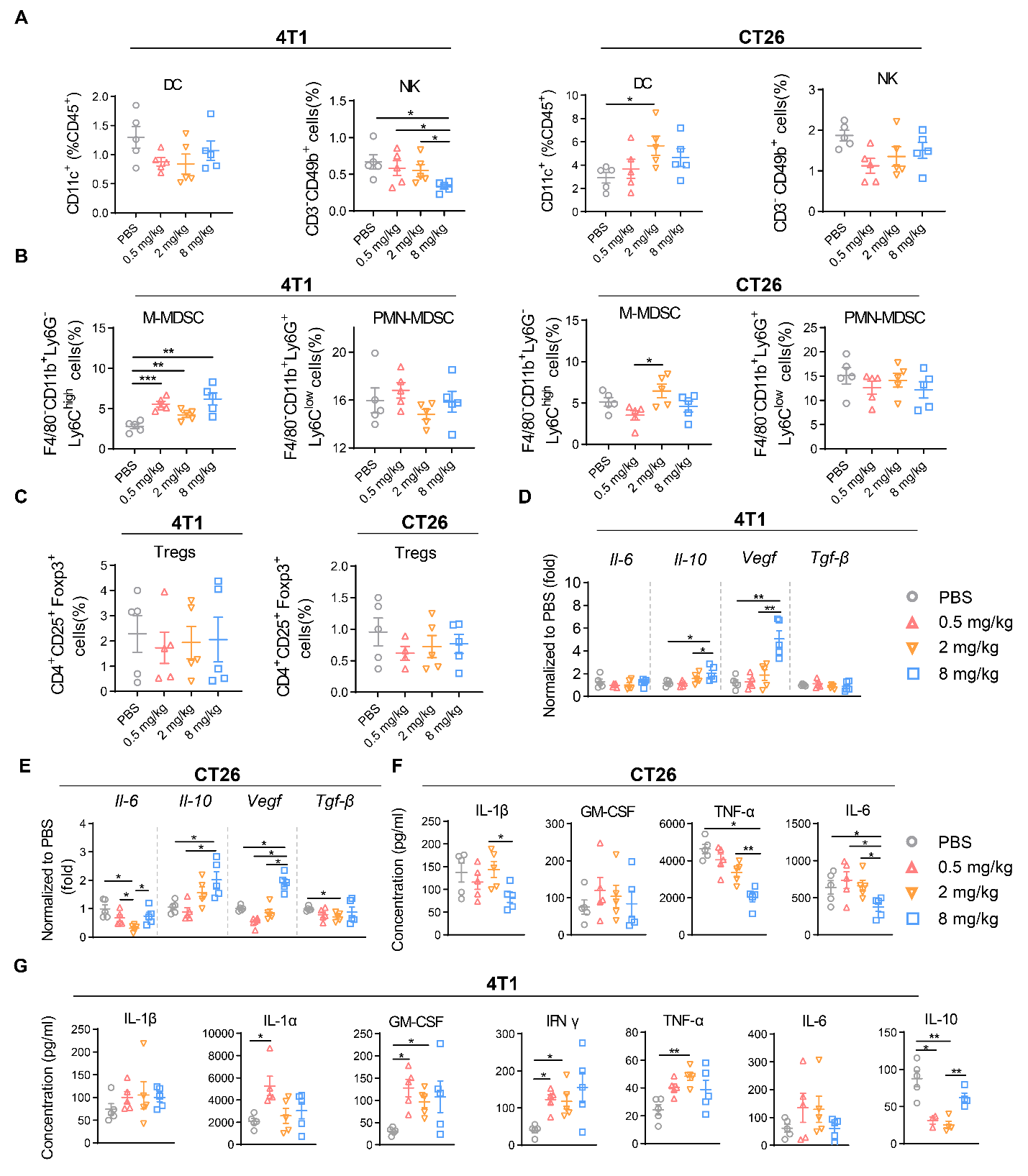
**Fig. S1.** **The percentage of immune cells and the expression of cytokines or chemokines in 4T1 and CT26 tumors.** **(A)** 4T1 and CT26 tumors were analyzed by flow cytometry to calculate the percentage of DC cells (CD45^+^CD11c^+^), activated DC cells (CD45^+^CD8^+^ CD69^+^), **(B)** M-MDSCs (F4/80^-^CD11b^+^Ly6G^-^Ly6C^high^), G-MDSC (F4/80^−^CD11b^+^Ly6G^+^Ly6C^low^), **(C)** Tregs (CD45^+^CD4^+^CD25^+^Foxp3). **(D and E)** Levels of mRNA expression of related cytokines and chemokines in 4T1 (D) or CT26 (E) tumors were analyzed by real-time RT-PCR and normalized to β-actin (n=5). **(F and G)** Mice bearing CT26 tumors(F) or 4T1 tumors (G) on day 12, the tumor tissues were obtained and the levels of cytokines, chemokines, and other meditators associated with tumor immunity were analyzed (n = 5). One-way ANOVA was performed to calculate the significant differences between groups, followed by LSD analysis. *P < 0.05; **P < 0.01.


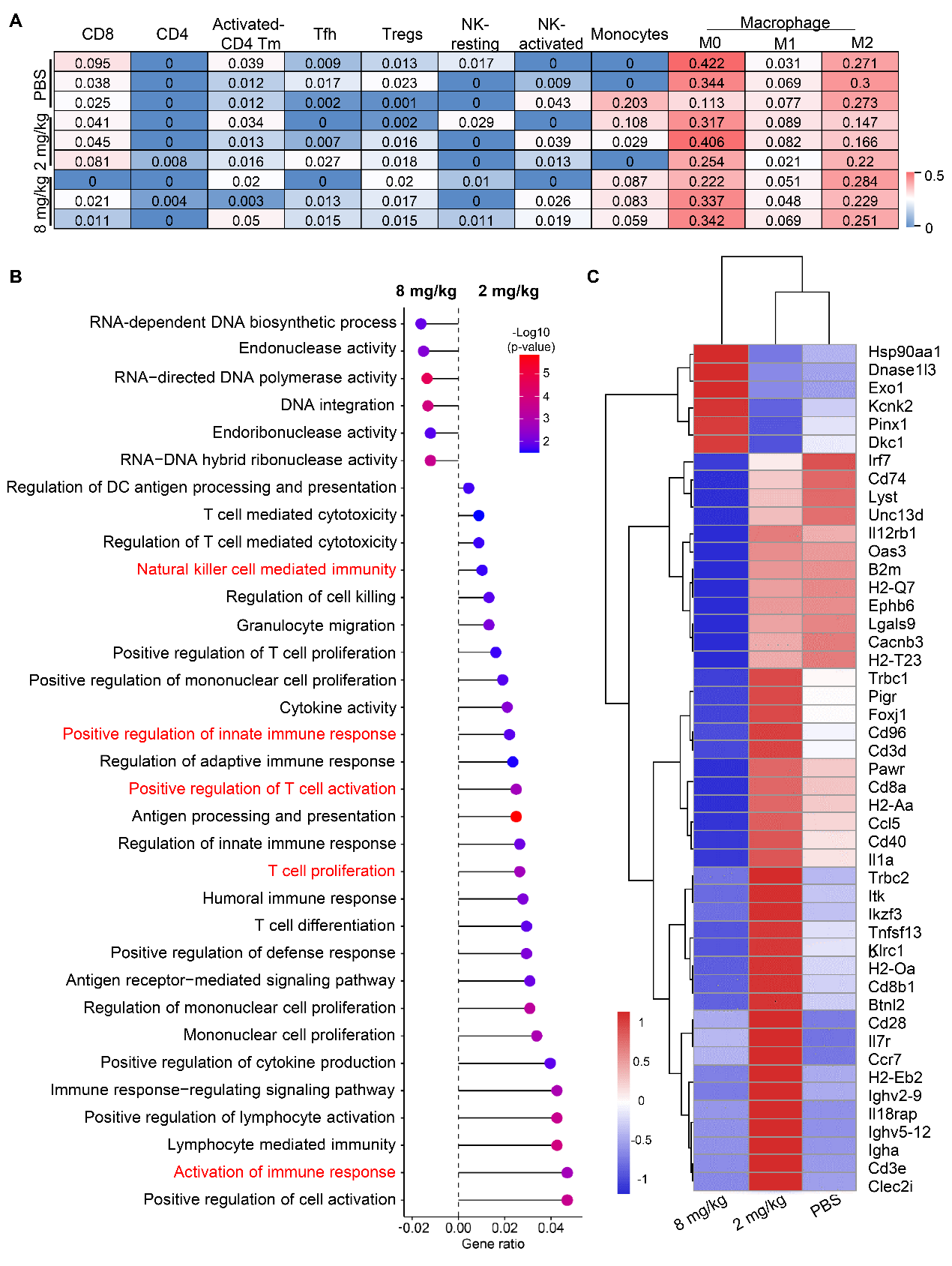


**Fig. S2. The RNA sequencing analysis of 4T1 tumor tissue after smTRAIL treatment. (A)** RNA-seq data from 4T1 tumor tissues treated with PBS, 2 mg/kg smTRAIL or 8 mg/kg smTRAIL were analyzed by CIBERSORT. Heat maps showing the proportion of immune cells, using the same colour settings. **(B)** Pathway enrichment analysis of 2 mg/kg versus 8 mg/kg smTRAIL treatment groups. **(C)** Heatmap of differential expression of key functional genes between PBS, 2 mg/kg smTRAIL and 8 mg/kg smTRAIL. Z-score normalized data is shown.


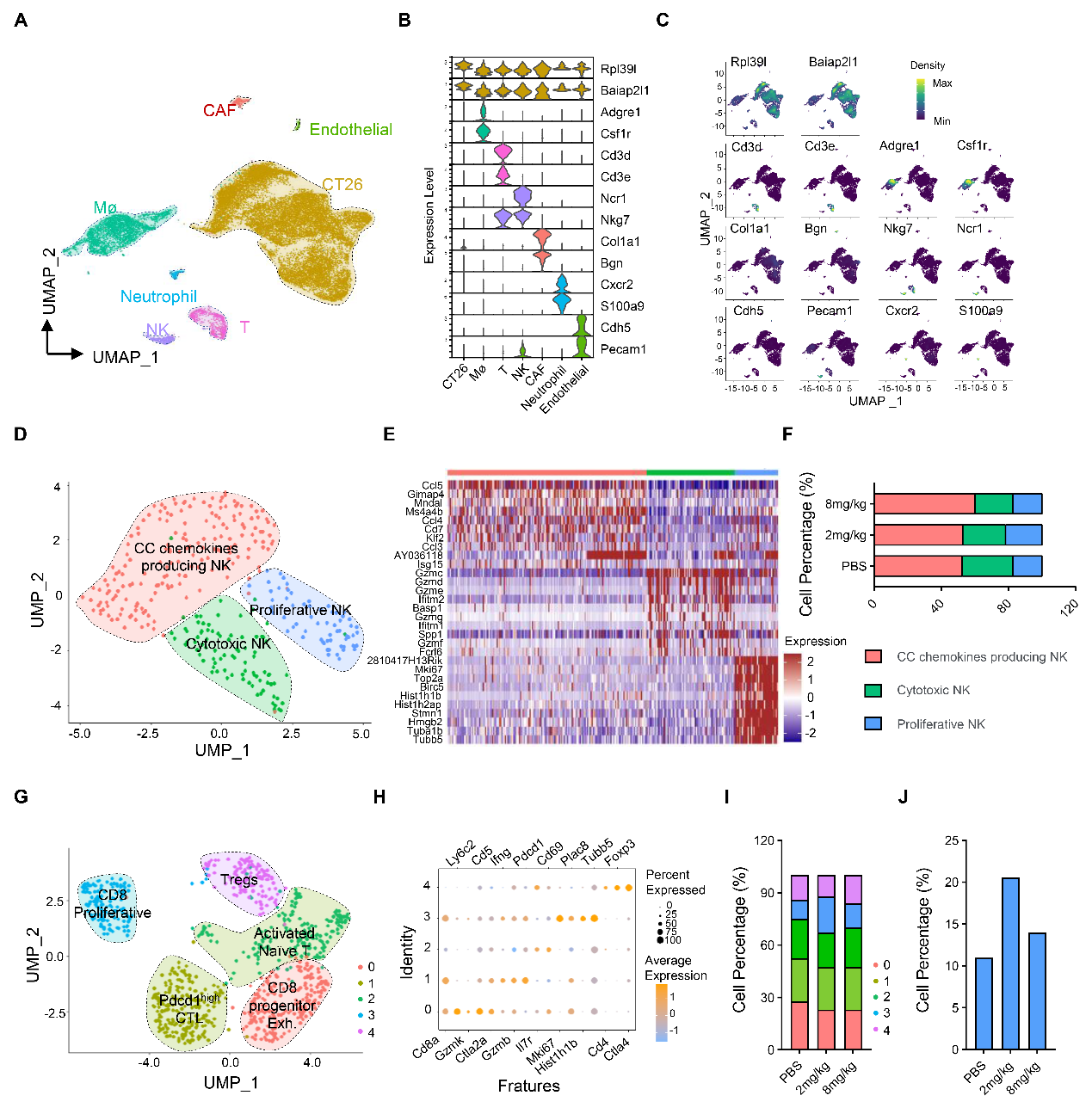


**Fig. S3. Characterization of tumor-infiltrating NK cells and CD8^+^ T cells by scRNA-seq in CT26 tumors. (A)** Uniform manifold approximation and projection (UMAP) visualization of cell clusters identified from scRNA-seq. **(B)** Expression of key genes used for identification of cell clusters from (A) by violin plot. **(C)** Expression levels of selected known marker genes using density plot. **(D)** UMAP visualization of reclustered NK cell from (A). **(E)** The heatmap shows key differentially expressed genes among NK cell populations identified in (D). Data are colored to reflect gene expression Z scores. **(F)** Stacked histograms of frequencies of clusters in groups. **(G)** UMAP visualization of reclustered T cell from (A). **(H)** Expression of key genes used for identification of cell clusters from (G) by bubble diagram. **(I)** Stacked histograms of frequencies of T cell clusters in groups. **(J)** The histograms of frequency of cluster 3 (proliferative CD8 T cell).


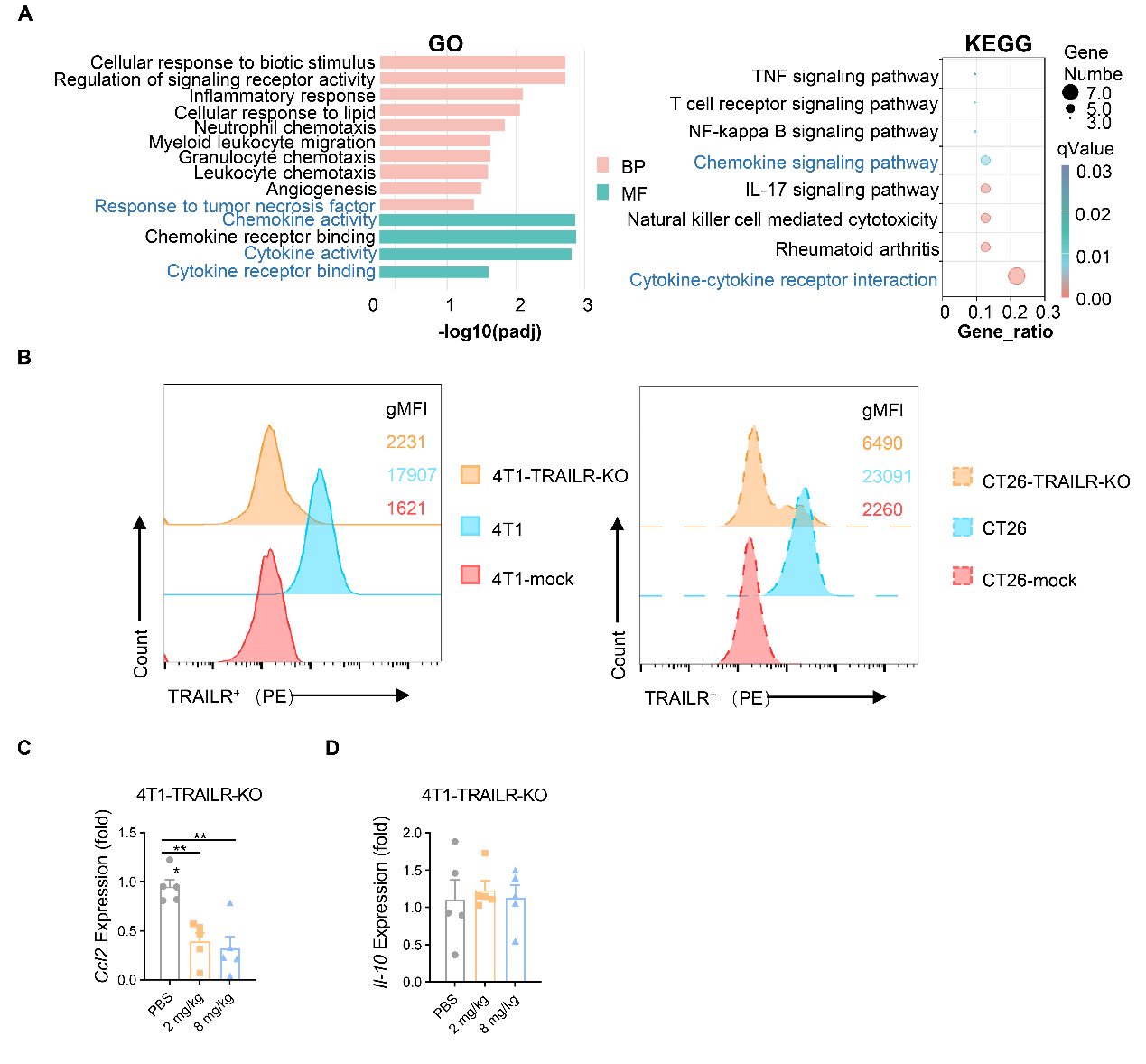


**Fig. S4. Exploring the mechanism of immunosuppressive microenvironment formation by tumor cells. (A)** GO analysis and KEGG analysis were shown associated with the expression of genes in Figure 5H. **(B)** Detection of TRAIL-R knock-out efficiency of two tumor cells by flow cytometry. **(C)** The levels of *Ccl2* mRNA and **(D)** *Il-10* mRNA in 4T1-TRAILR-KO tumor tissue were analyzed by real-time RT-PCR and normalized to β-actin (n=5). One-way ANOVA was performed to calculate the significant differences between groups, followed by LSD analysis. **P < 0.01.


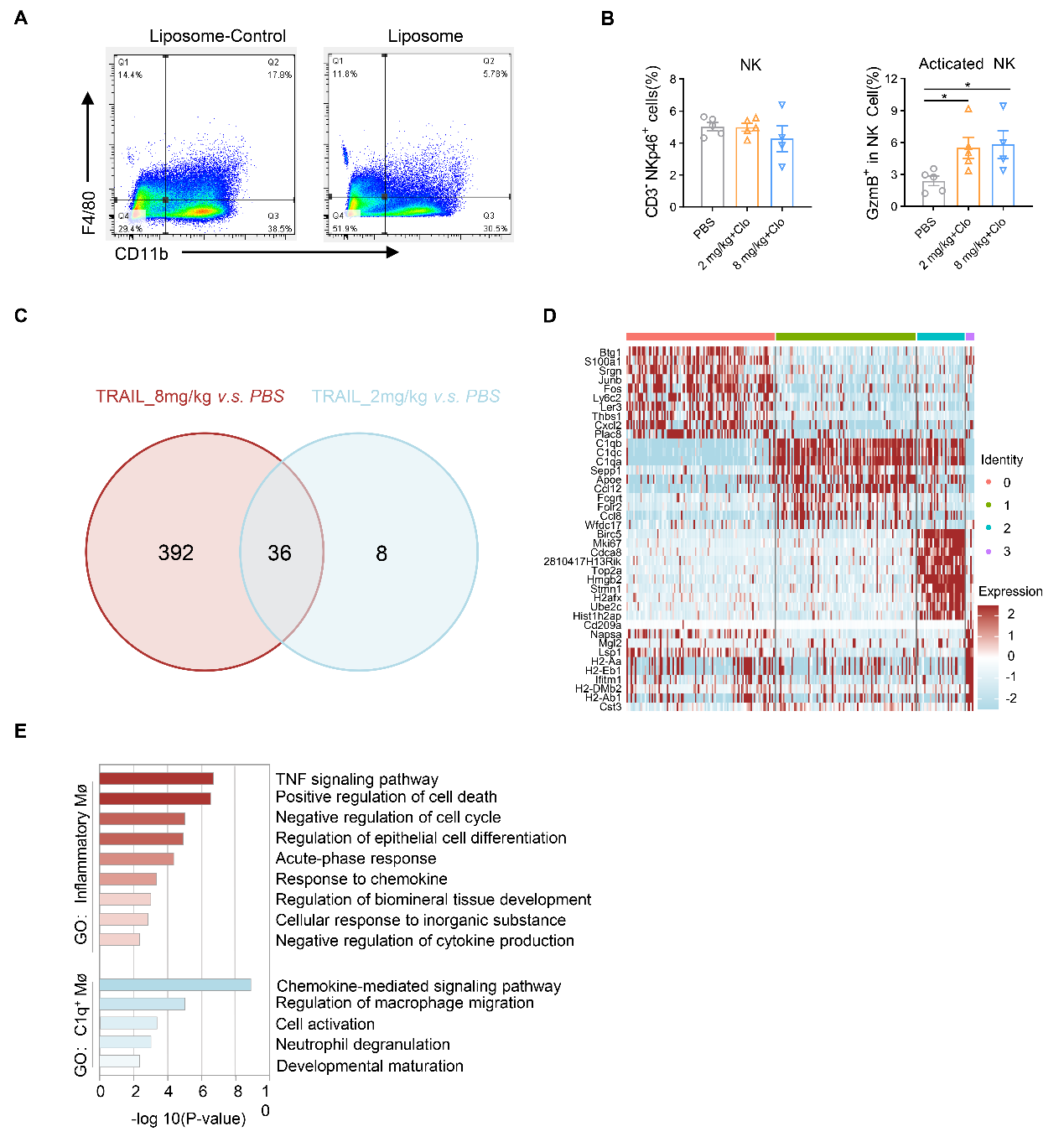


**Fig. S5. The evaluation of macrophage changes in vivo and the exploration of mechanism. (A)** After the treatment of clodronate liposomes the proportion of macrophage cells was evaluated. **(B)** Percentage of NK cells (CD3^-^CD49b^+^), activated NK cells (CD3^-^CD49b^+^CD107a^+^/GzmB^+^) were detected (n=5). **(C)** Venn diagram showing differentially expressed genes in macrophages in the TRAIL-treated and PBS groups. **(D)** Heatmap shows key differentially expressed genes among macrophage cell populations identified in Fig. **5C**. Data are colored to reflect gene expression Z scores. **(E)** Bar diagram displaying the enriched GO analysis of macrophage cluster C1q^+^ Mø and Inflammatory Mø. One-way ANOVA was performed to calculate the significant differences between groups, followed by LSD analysis. *P < 0.05.


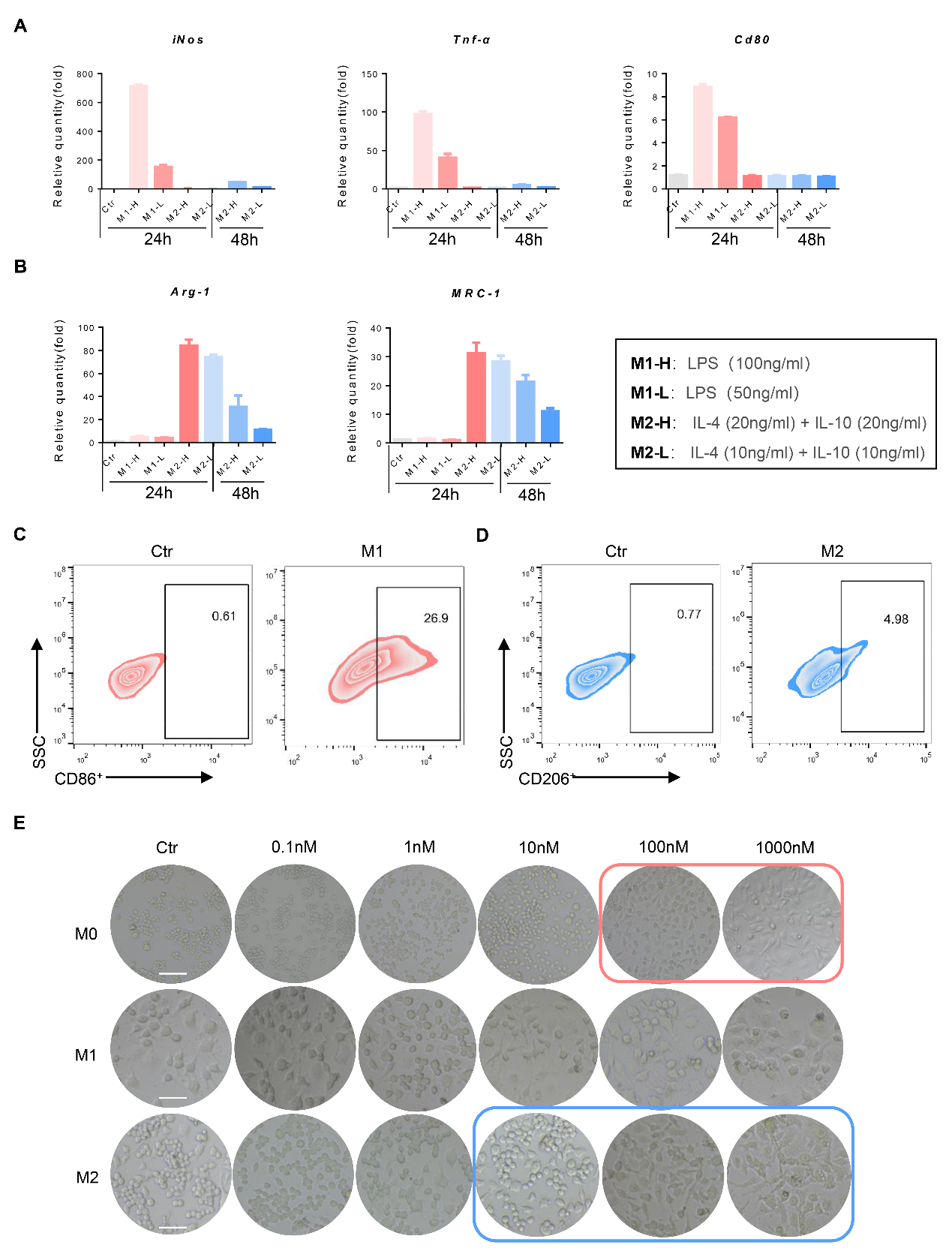


**Fig. S6. Polarization of macrophages in vitro by induction of cytokines. (A and B)** The levels of M1-related mRNA (A) and M2-related mRNA (B) were analyzed by real-time RT-PCR and normalized to β-actin (n=5). **(C and D)** Maker detection of M1-like (C) and M2-like (D) macrophages by flow cytometry. **(E)** The morphology of macrophage cells after incubation with different concentrations of smTRAIL. Scale bars, 50 μm.


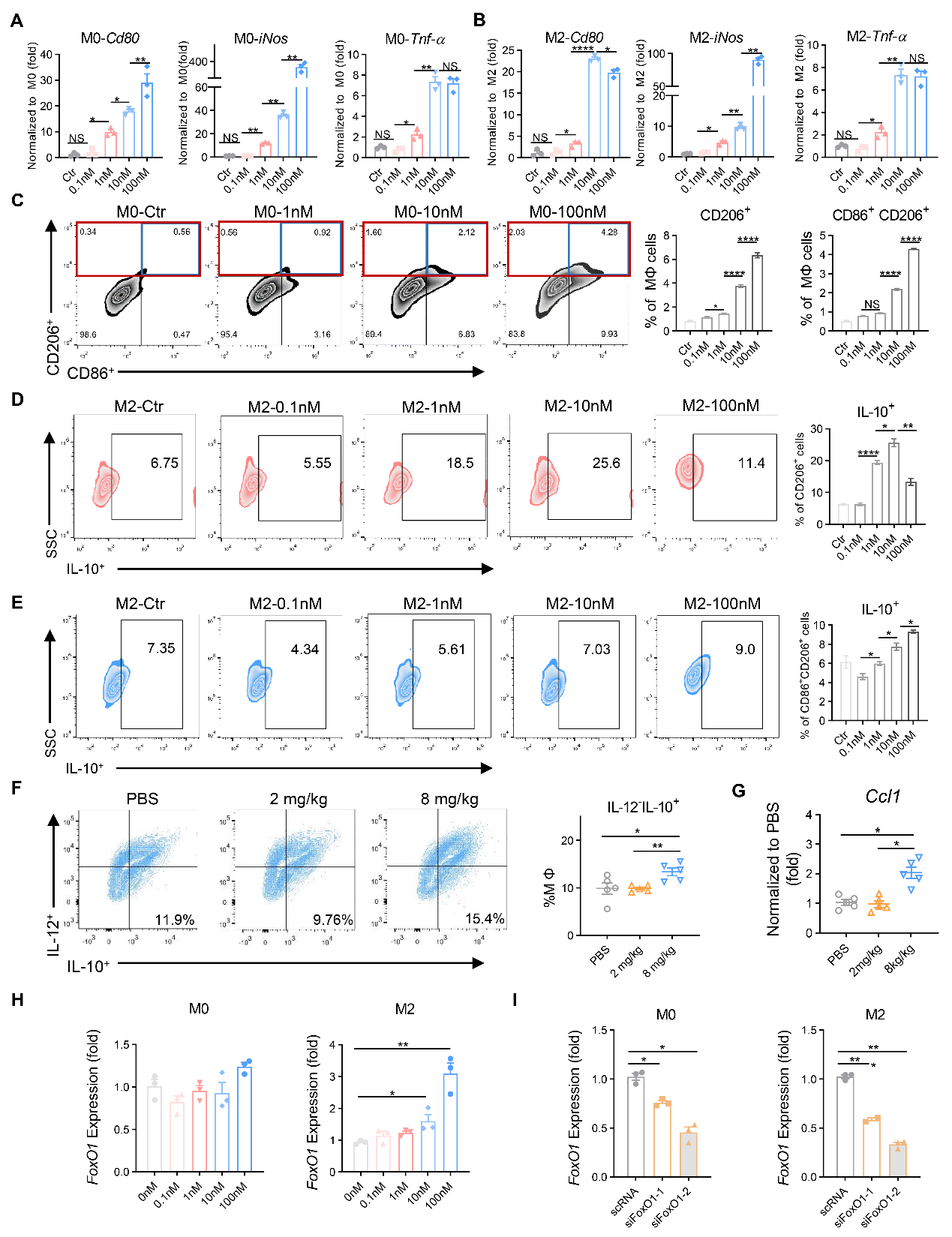


**Fig. S7. The effects of different concentrations of smTRAIL on macrophages. (A and B)** The levels of mRNA were analyzed by RT-PCR and normalized to β-actin (n=3). **(C)** The percentage of CD206^+^ M0-like macrophages (red frame) and CD86^+^CD206^+^ M0-like macrophages (blue frame) were detected by flow cytometry. **(D)** The percentage of IL-10^+^CD206^+^ in M2 incubated with smTRAIL (n=3) was detected by flow cytometry. **(E)** The percentage of IL-10^+^CD86^+^CD206^+^ in M2 incubated with smTRAIL (n=3) was detected by flow cytometry. **(F)** The percentage of M2b-like macrophages (IL-12^-^ IL-10^+^ macrophage) in CT26 tumor tissues was detected by flow cytometry (n=5). **(G)** Levels of mRNA expression of *Ccl1* in CT26 were analyzed by real-time RT-PCR and normalized to β-actin (n=5). **(H)** After M0 and M2-like macrophage cells were incubated with smTRAIL for 24 h, the levels of *Foxo1* mRNA were analyzed by real-time RT-PCR and normalized to β-actin (n=3). **(I)** The levels of *Foxo1* mRNA were analyzed by real-time RT-PCR and normalized to β-actin after silencing the FoxO1 gene in M0 and M2-like macrophage cells. (n=3).


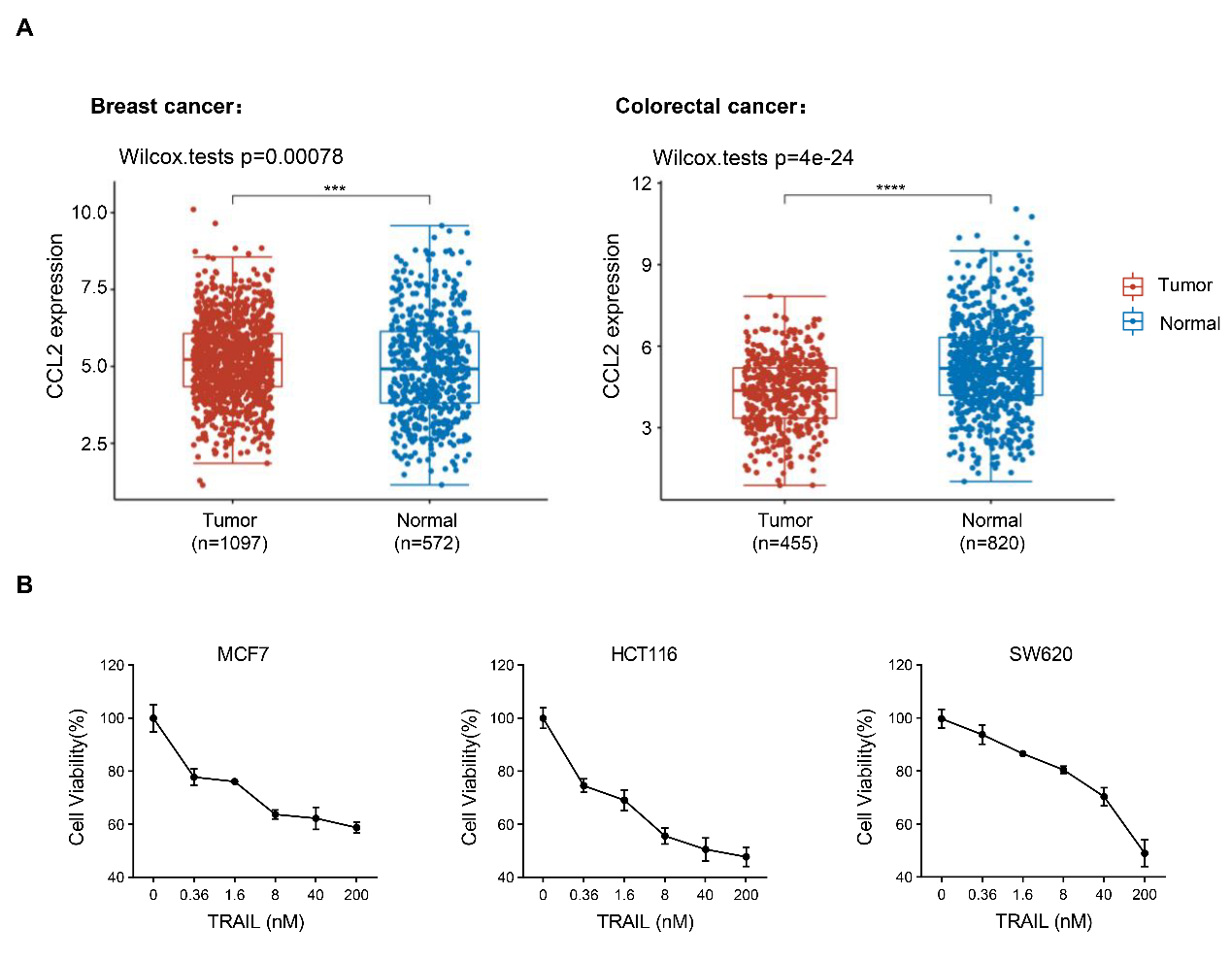


**Fig. S8. Evaluation of the killing ability of shTRAIL on human tumor cells. (A)** Comparison of CCL2 expression in breast or colorectal cancer tissues with normal tissues by TCGA database. **(B)** Three human cancer cells were treated with indicated concentrations of shTRAIL for 16h and cell viability was determined by MTT assay.

Table S1.

| **REGENT or RESOURCE** | **SOURCE** | **IDENTIFIER** |
| --- | --- | --- |
| **Antibodies** | | |
| FITC anti-mouse CD45 Antibody | BioLegend | Cat #: 103108; RRID: AB_312973 |
| PE/Cy7 anti-mouse CD335 (NKp46) Antibody | BioLegend | Cat #: 137618; RRID: AB_11219186 |
| PE/Cy5 anti-mouse CD3ε Antibody | BioLegend | Cat #: 100310; RRID: AB_312675 |
| APC/Cyanine7 anti-mouse CD49b Antibody | BioLegend | Cat #: 108920; RRID: AB_2561458 |
| PE anti-mouse CD107a (LAMP-1) Antibody | BioLegend | Cat #: 121611; RRID: AB_2134487 |
| PE anti-mouse CD253 (TRAIL) Antibody | BioLegend | Cat #: 109305; RRID: AB_2205927 |
| APC/Cy7 anti-mouse CD4 | BioLegend | Cat #: 100526; RRID: AB_312727 |
| APC anti-mouse CD8a Antibody | BioLegend | Cat #: 100712; RRID: AB_312751 |
| PE anti-mouse CD69 Antibody | BioLegend | Cat #: 104508; RRID: AB_313111 |
| APC anti-mouse CD25 Antibody | BioLegend | Cat #: 102012; RRID: AB_312861 |
| PE anti-mouse FOXP3 Antibody | BioLegend | Cat #: 126404; RRID: AB_1089117 |
| PE anti-mouse/human CD11b | BioLegend | Cat #: 101208; RRID: AB_312791 |
| PerCP/Cy5.5 anti-mouse Ly-6G | BioLegend | Cat #: 127616; RRID: AB_1877271 |
| PE/Cy7 anti-mouse CD11c | BioLegend | Cat #: 117318; RRID: AB_493568 |
| APC/Cy7 anti-mouse Ly-6C | BioLegend | Cat #: 128026; RRID: AB_10640120 |
| APC/Cy7 anti-mouse F4/80 | BioLegend | Cat #: 123118; RRID: AB_893477 |
| PerCP/Cy5.5 anti-mouse CD206 (MMR) | BioLegend | Cat #: 141716; RRID: AB_2561992 |
| PE/Cy7 anti-mouse CD16/32 | BioLegend | Cat #: 156610; RRID: AB_2800708 |
| PE/Cyanine7 anti-mouse CD45 Antibody | BioLegend | Cat #: 103114; RRID: AB_312979 |
| PE/Cyanine5 anti-mouse NK-1.1 Antibody | BioLegend | Cat #: 108716; RRID: AB_493590 |
| APC anti-mouse Perforin Antibody | BioLegend | Cat #: 154304; RRID: AB_2721463 |
| APC anti-mouse CD45 Antibody | BioLegend | Cat #: 103112; RRID: AB_312977 |
| PE/Cyanine7 anti-mouse CD80 Antibody | BioLegend | Cat #: 104734; RRID: AB_2563113 |
| APC/Cyanine7 anti-mouse CD69 Antibody | BioLegend | Cat #: 104526; RRID: AB_10679041 |
| FITC anti-mouse CD80 Antibody | BioLegend | Cat #: 104706; RRID: AB_313127 |
| PE anti-mouse IFNγ Antibody | BioLegend | Cat #: 505807; RRID: AB_315402 |
| PE anti-mouse CD86 Antibody | BioLegend | Cat #: 105008; RRID: AB_313151 |
| FITC anti-mouse CD4 Antibody | BioLegend | Cat #: 100510; RRID: AB_312713 |
| FITC anti-mouse CD3ε Antibody | BioLegend | Cat #: 100306; RRID: AB_312671 |
| PE anti-mouse CD253 (TRAIL) Antibody | BioLegend | Cat #: 109305; RRID: AB_2303575 |
| PE anti-mouse CD262 (DR5, TRAIL-R2) Antibody | BioLegend | Cat #: 400907; RRID: AB_326593 |
| PE/Cyanine7 anti-mouse IL-12 Antibody | BioLegend | Cat #: 505210; RRID: AB_2565645 |
| APC anti-mouse IL-10 Antibody | BioLegend | Cat #: 505010; RRID: AB_315364 |
| PE anti-human/mouse Granzyme B Antibody | BioLegend | Cat #: 372208; RRID: AB_2687032 |
| 488-conjugated CD206 Monoclonal antibody | Proteintech | Cat #: CL488-60143; RRID: AB_2883105 |
| primary goat anti-cleavage caspase-3 | Cell Signaling Technology | Cat #: 9661 |
| Anti Asialo GM1 Antibodies | FUJIFILM Wako | Cat #: 986-10001; RRID: AB_516844 |
| TruStain FcX™ (anti-mouse CD16/32) Antibody | BioLegend | Cat #: 101320; RRID: AB_1574975 |
| **Chemicals, peptides, and recombinant proteins** | | |
| Soluble murine TRAIL (smTRAIL) | Xupu Wang et al. ^1^ | N/A |
| Soluble human TRAIL (shTRAIL) | J Yan et al. ^2^ | N/A |
| RBC Lysis Buffer (10X) | BioLegend | Cat #: 420301; |
| Fixation/Permeabilization concentrate | eBioscience | Cat #: 00-5123-43 |
| eBioscience^TM^ Fixation/perm diluent | eBioscience | Cat #: 00-5223-56 |
| Permeabilization Buffer 10x | eBioscience | Cat #: 00-8333-56 |
| collagenase | Roche | Cat #: 5401054001 |
| Dnase Ⅰ | Sigma | Cat #: D5025 |
| recombinant mouse IL-2 | Peprotech | Cat #: 212-12-5μg |
| LPS | Sigma | Cat #: L3129-10MG |
| recombinant mouse IL-4 | Peprotech | Cat #: 214-14-5μg |
| recombinant mouse IL-10 | Peprotech | Cat #: 210-10-2μg |
| DAPI | Sigma | Cat #: D9542 |
| Combo: Clophosome®-A and Control Liposomes | FormuMax | Cat #: F70101C-AC |
| **Critical Commercial Assays** | | |
| TransZol^TM^ Up Plus RNA Kit | TransGen Biotech | Cat #: ER501 |
| PrimeScript^TM^ 1^st^ Strand cDNA Synthesis Kit | TaKaRa Biotech | Cat #: 6110A |
| NK cell isolation kit | Miltenyi Biotec | Cat #: 130-115-818 |
| Lactate dehydrogenase cytotoxicity assay kit | Beyotime | Cat #: C0017 |
| LEGENDplex™ Mouse Inflammation Panel (13-plex) with Filter Plate | BioLegend | Cat #: 740150 |
| DAB Detection Kit | MXB Biotechnologies | Cat #: DAB-1031 |
| Rabbit Two-Step Kit | ZSGB-BIO | Cat #: PV-6001 |
| **Oligonucleotides** | | |
| Mouse *β-actin* qRT-PCR F  CTCTGTGTGGATCGGTGGC (Tm: 61.9℃) | This paper | N/A |
| Mouse *β-actin* qRT-PCR R  GTAAAACGCAGCTCAGTAACAGTC (Tm: 60.3℃) | This paper | N/A |
| Mouse *Il-6* qRT-PCR F  TCCAGTTGCCTTCTTGGGAC (Tm: 59.8℃) | This paper | N/A |
| Mouse *Il-6* qRT-PCR R  GTGTAATTAAGCCTCCGACTTGTG (Tm: 57.6℃) | This paper | N/A |
| Mouse *Il-10* qRT-PCR F  CAGGGATCTTAGCTAACGGAAA (Tm : 58.5℃) | This paper | N/A |
| Mouse *Il-10* qRT-PCR R  GCTCAGTGAATAAATAGAATGGGAAC (Tm : 55.8℃) | This paper | N/A |
| Mouse *Tnf-α* qRT-PCR F  ACAGAAAGCATGATCCGCGA (Tm : 61.4℃) | This paper | N/A |
| Mouse *Tnf-α* qRT-PCR R  TGGGGACCGATCACCCC (Tm : 63.5℃) | This paper | N/A |
| Mouse *Ccl2* qRT-PCR F  TGACCCCAAGAAGGAATGGG (Tm:59.9℃) | This paper | N/A |
| Mouse *Ccl2* qRT-PCR R  ACCTTAGGGCAGATGCAGTT (Tm:57.8℃) | This paper | N/A |
| Mouse *Ifn-γ* qRT-PCR F  TCAAGTGGCATAGATGTGGAAGAA (Tm : 59.9℃) | This paper | N/A |
| Mouse *Ifn-γ* qRT-PCR R  TGGCTCTGCAGGATTTTCAT (Tm : 58℃) | This paper | N/A |
| Mouse *Vegf* qRT-PCR F  CGCCGCAGGAGACAAACCGAT (Tm : 69℃) | This paper | N/A |
| Mouse *Vegf* qRT-PCR R  ACCCGTCCATGAGCTCGGCT (Tm : 66.3℃) | This paper | N/A |
| Mouse *Tgf-β* qRT-PCR F  TCCCCTCCGAAAATGCCATC (Tm : 64.3℃) | This paper | N/A |
| Mouse *Tgf-β* qRT-PCR R  GAAGACCCTGAACTCTGCCTT (Tm : 57.6℃) | This paper | N/A |
| Mouse *iNos* qRT-PCR F  CAATGGCAACATCAGGTCGG (Tm:59.9℃) | This paper | N/A |
| Mouse *iNos* qRT-PCR R  CGTACCGGATGAGCTGTGAA (Tm:59.9℃) | This paper | N/A |
| Mouse *Arg-1* qRT-PCR F  GTACATTGGCTTGCGAGACG (Tm:59.9℃) | This paper | N/A |
| Mouse *Arg-1* qRT-PCR R  GTACATTGGCTTGCGAGACG (Tm:57.8℃) | This paper | N/A |
| Mouse *Mrc-1* qRT-PCR F  GGATTGCCCTGAACAGCAAC (Tm:59.9℃) | This paper | N/A |
| Mouse *Mrc-1* qRT-PCR R  ACTTAAGCTTCGGCTCGTCA (Tm:57.8℃) | This paper | N/A |
| Mouse *Cd80* qRT-PCR F  GGCAAGGCAGCAATACCTTA (Tm:57.8℃) | This paper | N/A |
| Mouse *Cd80* qRT-PCR R  CTCTTTGTGCTGCTGATTCG (Tm:57.8℃) | This paper | N/A |
| Mouse *FoxO1* qRT-PCR F  CTGTGACATGGAGTCCATCA (Tm:57.8℃) | This paper | N/A |
| Mouse *FoxO1* qRT-PCR R  TGTAGCCTGCTCACTAACTC (Tm:57.3℃) | This paper | N/A |
| Human *Gapdh* qRT-PCR F  AGGTCGGAGTCAACGGAT (Tm: 57.3℃) | This paper | N/A |
| Human *Gapdh* qRT-PCR R  TCCTGGAAGATGGTGATG (Tm: 55℃) | This paper | N/A |
| Human *Ccl2* qRT-PCR F  CTCGCCTCCAGCATGAAAGT (Tm: 59.9℃) | This paper | N/A |
| Human *Ccl2* qRT-PCR R  AGATCTCCTTGGCCACAATGG (Tm: 60℃) | This paper | N/A |
| Mouse TRAILR gRNA1-top  CACCGGTGGGCGTGCTGGGTCCTGG (Tm: 73.5℃) | This paper | N/A |
| Mouse TRAILR gRNA1-bottom  AAACCCAGGACCCAGCACGCCCACC (Tm: 70.2℃) | This paper | N/A |
| Mouse TRAILR gRNA2-top  CACCGATCGTCCAGCTGGCCTACAG (Tm: 68.5℃) | This paper | N/A |
| Mouse TRAILR gRNA2-bottom  AAACCTGTAGGCCAGCTGGACGATC (Tm: 65.3℃) | This paper | N/A |
| **Experimental model: Organisms/strains** | | |
| C57/BL6J mice | Liaoning Changsheng Biotechnology | N/A |
| BALB/c mice | Liaoning Changsheng Biotechnology | N/A |
| BALB/c – nude mice | Beijing Vital River Laboratory Animal Technology | N/A |
| huHSC-NPG-GM3 | Beijing Vitalstar Biotechnology | N/A |
| **Software and algorithms** | | |
| R 4.2.2 | The Comprehensive R Archive Network | https://cran.r-project.org/ |
| RStudio1.4.1717 | RStudio | https://www.rstudio.com/ |
| Seurat_4.1.0 | Phan et al.^3^ | https://github.com/satijalab/seurat |
| GSEA 4.2.3 | Barnden et al.^4^ | https://www.gsea-msigdb.org/gsea |
| Harmony | Korsunsky et al.^5^ | https://github.com/immunogenomics/harmony |
| ggplot2 | Wickham et al.^6^ | https://github.com/tidyverse/ggplot2 |
| clusterProfiler | Guangchuang Yu et al.^7^ | https://github.com/YuLab-SMU/clusterProfiler |
| msigdbi | Liberzon A et al.^8^ |  |
| Nebulosa | Mehta, R.S. et al. ^9^ | https://github.com/powellgenomicslab/Nebulosa |
| sctransform | Choudhary, S. et al.^10^ | https://github.com/satijalab/sctransform |
| Cell Ranger | 10x Genomics | https://support.10xgenomics.com/single-cell-gene-expression/software/downloads/ latest |
| Flowjo, version 10 | Tree Star | https://www.flowjo.com/ |
| Prism, version 8 | GraphPad | https://www.graphpad.com/ scientific-software/prism/ |
| Adobe Photoshop | Adobe | https://www.adobe.com/au/products/ photoshop.html |

**Reference**

1. Wang X, Wang L, Liu W, Feng X, Wu H, Zhang H, et al. Production of soluble murine TRAILs in Escherichia coli with Zn2+ supplementation. Protein Pept Lett. 2022.

2. Yan J, Wang L, Wang Z, Wang Z, Wang B, Zhu R, et al. Engineered adenovirus fiber shaft fusion homotrimer of soluble TRAIL with enhanced stability and antitumor activity. Cell Death & Disease. 2016;7.

3. Tri Giang P, Michelle A, Sandra G, Jeffrey C, Jhagvaral H, Hodgkin PD, et al. B cell receptor-independent stimuli trigger immunoglobulin (Ig) class switch recombination and production of IgG autoantibodies by anergic self-reactive B cells2018.

4. Barnden MJ, Allison J, Heath WR, Carbone FR. Defective TCR expression in transgenic mice constructed using cDNA-based alpha- and beta-chain genes under the control of heterologous regulatory elements. Immunology and cell biology. 1998;76: 34-40.

5. Korsunsky I, Millard N, Fan J, Slowikowski K, Raychaudhuri S. Fast, sensitive and accurate integration of single-cell data with Harmony. Nature Methods. 2019;16: 1-8.

6. Wickham H. Ggplot2: Elegant Graphics for Data Analysis. ggplot2: Elegant Graphics for Data Analysis, 2009.

7. Yu G, Wang LG, Han Y, He QY. clusterProfiler: an R package for comparing biological themes among gene clusters. Omics-a Journal of Integrative Biology. 2012;16: 284-287.

8. Mesirov JP. Molecular signatures database (MSigDB) 3.0. Bioinformatics. 2011;27: 1739.

9. Mehta RS, Donohoe KR. Snowflake morays, Echidna nebulosa , exhibit similar feeding kinematics in terrestrial and aquatic treatments. Journal of Experimental Biology. 2021;224.

10. Choudhary S, Satija R. Comparison and evaluation of statistical error models for scRNA-seq. Cold Spring Harbor Laboratory. 2021.
